# Blood Profiling of Athletes after COVID-19: Differences in Blood Profiles of Post-COVID-19 Athletes Compared to Uninfected Athletic Individuals—An Exploratory Analysis

**DOI:** 10.3390/biomedicines11071911

**Published:** 2023-07-06

**Authors:** Shirin Vollrath, Lynn Matits, Achim Jerg, Jule Zorn, Lucas John, Jürgen Michael Steinacker, Daniel Alexander Bizjak

**Affiliations:** 1 Division of Sports and Rehabilitation Medicine, Department of Medicine, Ulm University Hospital, 89075 Ulm, Germany; lynn.matits@uni-ulm.de (L.M.); achim.jerg@uniklinik-ulm.de (A.J.); jule.zorn@tum.de (J.Z.); lucas.john@tum.de (L.J.); juergen.steinacker@uniklinik-ulm.de (J.M.S.); daniel.bizjak@uniklinik-ulm.de (D.A.B.); 2 Division of Clinical & Biological Psychology, Institute of Psychology and Education, Ulm University, 89075 Ulm, Germany

**Keywords:** blood analysis, COVID-19, inflammation, physical activity, athletes, reference values, long-term changes, sex differences

## Abstract

Blood profiling data in athletic populations and their respective responses to SARS-CoV-2 infection are lacking. Thus, this exploratory pilot study aimed to analyze and compare clinical blood markers in previously infected trained athletes (ATH; 30 m/29 f) and a not previously infected healthy athletic control group (HC; 12 m/19 f). The ATH group undertook a sports medical examination which included extended blood analyses. Blood profiles with a total of 74 variables were assessed (blood counts, pro-/inflammatory and immunological markers, and micronutrients), and the ATH group was compared to the age-matched, vaccinated HC group with comparable athletic back grounds, though without previous SARS-CoV-2-infections. The ATH group showed lower IgG, Troponin-T levels, and they had a lower complement/acute-phase protein activation. Furthermore, Vitamin D levels were lower and electrolyte/micronutrient concentrations were higher in ATH. Soluble transferrin receptor as a marker of erythrocyte turnover was decreased whereas PTT as a coagulation marker was increased. Subgroup analyses according to sex revealed more differences between the women of the ATH and HC groups (for 25 different variables) than between the men (for 5 different variables), especially for immunological and metabolic variables. In particular, the immune system and electrolyte/micronutrient status should be observed frequently and sex-specifically in this athletic cohort.

## 1. Introduction

SARS-CoV-2 infection can lead to several long-lasting complications which have been summarized by post-COVID studies and can have different physiological and psychological outcomes [1]. Symptoms include fatigue, general pain or discomfort, attention disorders, sleep disturbances, hair loss, shortness of breath, and anxiety or depression, with up to 32 additional different complications having been observed [2,3]. The sheer quantity of these volatile symptoms underlines that there is not one “common denominator” causing the long-term adverse health effects. The proposed mechanisms include a hyperinflammatory immune response, a so-called cytokine storm, mitochondrial damage, and decreased red blood cell rheological function [4,5,6,7,8]. Furthermore, it has been shown that altered blood parameters correlate with depressive symptoms and that an individual’s perception of recovery may be impaired over a longer period of time after COVID-19 infection [9,10]. The different virus variants, as well as individual health and vaccine statuses, make the prognosis of disease severity even more difficult. Therefore, robust and valid clinical blood or saliva markers would be very useful for healthcare advisers/physicians for monitoring the health statuses of their patients or for intervening to reduce the detrimental results of SARS-CoV-2 infections.

Although the positive health effect of regular physical exercise on immune function is well known [11], the “athletic population” compromises only a minor percentage of the world-wide population [12]. As consequence, the pandemic has led to medical studies mostly focusing on the general population, which has tendencies toward highly sedentary lifestyles, obesity, and cardiovascular disease burdens, and this generalizes study results for COVID-19 without discriminating between regular exercising individuals and sedentary ones. However, the responses observed in athletes may reveal phylogenetic—currently defined “normal”—human responses after a SARS-CoV-2 infection, while routinely used thresholds of clinical blood markers and cardiovascular features such as heart frequency differ in athletes. This may result in medical advice that is not suitable or relevant for this specific population [13,14,15].

Blood profiles in acutely infected COVID-19-patients without comorbidities have shown that the blood markers interleukin-6 (IL-6) and tumor-necrosis-factor-alpha (TNF-α) are strong predictors for disease severity and death [16], while there are also non-cytokine biomarkers such D-dimers, C-reactive protein (CRP), and ferritin that are also elevated in COVID-19-infected patients compared to non-COVID-19-infected people [17,18].

Furthermore, increased T-cell activation has been observed and is indicated by the level of soluble interleukin IL-2 receptor (sIL-2R), regardless of age, in children and adults with severe COVID-19 infection [19]. Regarding the virus variants before Omicron, Conti et al. observed that in addition to the cytokine IL-6, IL-1β contributes to disease severity by driving inflammation [20].

As all these results have been determined in different study populations without connections to physical activity level and regular exercise, and the possible transfer of these data to individuals with high degrees of physical fitness is still largely unknown.

Thus, the aim of this pilot study was to examine the blood profiles of physically active individuals post-COVID-19 infection and compare them with the blood levels of non-infected control athletes to identify abnormalities in blood markers due to COVID-19.

## 2. Materials and Methods

### 2.1. Recruitment

Participants were recruited from consecutive athletic patients after previous SARS-CoV-2 infection at the Department of Sports and Rehabilitation Medicine, University Ulm, during March 2020 and July 2021, and they provided informed consent to participate in extended blood profiling. Some individuals were also part of the national study CoSmo-S (COVID-19 in German Competitive Sports [21]) while attending physical examinations after COVID-19 infection for the evaluation of their exercise capacity and physical resilience. Subjects in the age-matched healthy control group were recruited during presentations for their annual routine check-ups at the same facility, but they were not participating in another study and their clinical data were analyzed retrospectively. To avoid inclusion of individuals with a previous, non-detected subclinical SARS-CoV-2 infection in the control group, the analysis included mainly individuals with at least two to three negative SARS-CoV-2 PCR tests over the preceding three months before inclusion.

### 2.2. Study Population

The inclusion criteria for the enrolled participants were: athletes aged ≥ 18 years with a previous SARS-CoV-2 infection and a proof of such previous infection by (1) positive SARS-CoV-2 PCR or (2) antibody detection against SARS-CoV-2 with typical symptoms and (3) extensive blood profiling during the study visits. Athletes were defined as such if they exercised at least three times per week, with a metabolic equivalent of >20 h/week (e.g., cycling at 20 km/h = 7.1 metabolic equivalents [22]). Athletes with a positive SARS-CoV-2 PCR test within the preceding two weeks, insufficient German language skills, refusal of venous blood collection, and current diseases (acute and chronic) that did not allow for admission as estimated by the study physician were excluded [21].

The blood profiles of the included athletes (ATH) were compared to the blood profiles of age-matched, vaccinated, healthy controls (HC; N = 31, 12 m/19 f, aged 31.1 ± 10.4 years, body mass of 72.8 ± 13.9 kg, body height of 176.1 ± 8.93 cm, and BMI of 23.4 ± 3.5 kg/m^2^) without prior SARS-CoV-2 infection and fitness statuses as well as anthropometric variables (sex, age, body mass, height, and BMI) that were comparable to those of the ATH group. Table 1 shows detailed information about the study population.

### 2.3. Blood Sampling and Analysis

For the blood profiling, 9 mL of EDTA-anticoagulated and 9 mL of serum blood samples (Sarstedt, Nümbrecht, Germany) were taken from veins in the participants’ forearms. Blood sampling was conducted before the patients were physically active. Fasting state and sampling in the morning were preferred, but there were no prerequisites for sampling. In total, 74 individual blood variables were determined and analyzed by clinical standards with a DXH Coulter or a Sysmex system (based on resistance measurement principles (impedance measurements and the Coulter measurement principle), photometric measurements, and differentiation in flow cells by means of laser via VCSn technology (volume, conductivity, and scatter)), as well as ECLIA (Roche Immunoassay Analyzer Cobas 8000, Cobas t 711, and Cobas t 511, Rotkreuz, Suisse) and ELISA measurements.

For providing a reasonable overview, we grouped the respective 74 variables in the following subcategories: (1) blood cell system, (2) inflammation/immunology, (3) coagulation, (4) damage markers, (5) electrolytes/micronutrients, and (6) metabolism.

### 2.4. Statistical Analysis

Data analysis was performed with R, version 4.1.1 (2021-08-10; Non Profit/Open Access), and IBM SPSS Statistics, version 28.0.0.0 (IBM Deutschland GmbH, Ehningen, Germany). The graphics and figures were created with GraphPad PRISM (Version 9.3, La Jolla, CA, USA). To investigate whether there were differences in the blood parameters of athletes who suffered from COVID-19 compared to the fit healthy controls, two-tailed unpaired Wilcoxon tests were conducted. To detect possible differences between the anthropometric data and the training volumes before COVID-19 infection, Mann–Whitney U-tests and a Pearson chi-square test were conducted. Robust linear regression models were performed to analyze the influence of the time that had passed between infection and blood-taking on the concentrations of the blood parameters (R-package: “Robust” [23]). For IL-1β, IL-6, IL-8, IL-10, TNF-α, CRP, Troponin-T, Folic acid, D-dimers, and CH50, values below the limit of detection were replaced by half of the corresponding detection limit. Separately conducted subgroup-analyses for the control variables (age, sex, and time passed between infection and blood-taking) were performed additionally. For all analyses, an α-level of 0.050 was considered as significant. The adjusted significance level was *p* < 0.0007 when considering multiple testing (Bonferroni correction). All data are presented as means ± standard deviations if not otherwise stated.

### 2.5. Ethics, Consent, and Permissions

All participating athletes took part voluntarily and provided informed consent prior to inclusion. The study was performed in accordance with the Declaration of Helsinki. The study was approved by the ethics committee of Ulm University (EK 408/20). The study has been registered in the German Clinical Trials Register (DRKS00023717). The study reporting adheres to the CONSORT guidelines for reporting clinical trials.

## 3. Results

### 3.1. Blood Variables: Comparison between the Athletes and the Healthy Athletic Control Group

#### 3.1.1. Blood Cell System

**Figure 1:**
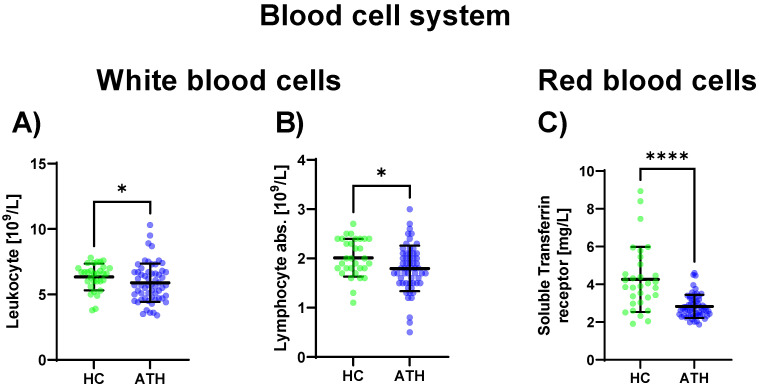
Blood cell system-specific differences between the previously COVID-19-infected athletes (ATH) and the healthy controls (HC) with comparable fitness statuses. Of the 27 variables grouped in the blood cell system, in the sub-group of white blood cells, significant differences for (**A**) leucocyte (HC 6.3 ± 1.0 vs. ATH 5.9 ± 1.5 $10^{9}$/L) and (**B**) absolute lymphocyte (HC 2.0 ± 0.4 vs. ATH 1.8 ± 0.5 $10^{9}$/L) concentrations were observed. In the sub-group of red blood cells, the specific variable (**C**) soluble transferrin receptor (HC 4.3 ± 1.7 vs. ATH 2.8 ± 0.6 mg/L) concentration was significantly different between the ATH and HC groups. * *p* ≤ 0.05 and **** *p* ≤ 0.0001 for the ATH group compared to the HC group.

Regarding the sub-group of white blood cells, the leukocyte concentration was decreased in the ATH group compared to the HC group (*p* = 0.034), as well as the absolute lymphocyte concentration (*p* = 0.023) which was also decreased in the ATH group. Regarding differences in the red blood cell system, the soluble transferrin receptor (*p* < 0.001) showed decreased values in the ATH group compared to the HC group (Figure 1).

#### 3.1.2. Inflammation/Immunology

**Figure 2:**
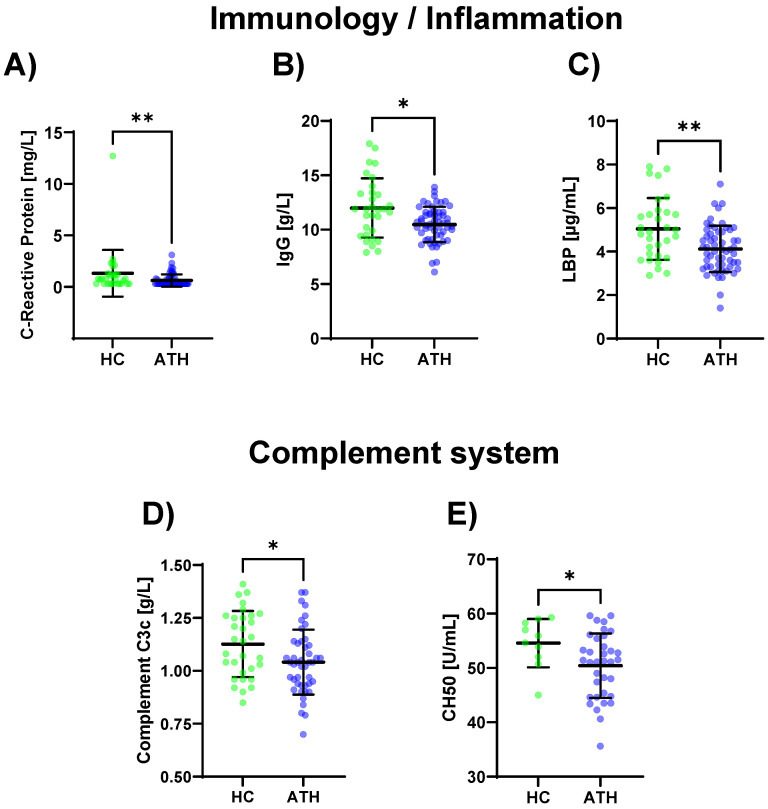
Differences in the variables regarding immunology/inflammation (**A**–**C**) and complement system (**D**,**E**) between the previously infected athletes (ATH) and the healthy controls (HC) with comparable fitness statuses. Of the 17 variables grouped for inflammation/immunology, the (**A**) CRP (HC 1.3 ± 2.2 vs. ATH 0.6 ± 0.6 mg/L), (**B**) IgG (HC 12.0 ± 2.7 vs. ATH 10.5 ± 1.6 g/L), and (**C**) LBP (HC 5.0 ± 1.4 vs. ATH 4.1 ± 1.1 μg/mL) concentrations were significantly different between the ATH and HC groups. The levels of (**D**) complement C3c (HC 1.1 ± 0.2 vs. ATH 1.0 ± 0.2 g/L) and (**E**) CH50 (HC 54.6 ± 4.4 vs. ATH 50.4 ± 5.9) and sub-grouped from immunology, differed between the ATH and HC groups. * *p* ≤ 0.05 and ** *p* ≤ 0.01 for the ATH group compared to the HC group.

Lower values for the SARS-CoV-2 spike antibody (*p* < 0.001), CRP (*p* = 0.012), IgG (*p* = 0.013), and LBP (*p* = 0.004) were determined for the ATH group compared to the HC group (Figure 2A–C Table S1). The complement-specific analyte CH50 (*p* = 0.045) and complement C3c (*p* = 0.030) showed decreased values in the ATH group (Figure 2D,E).

#### 3.1.3. Coagulation

**Figure 3:**
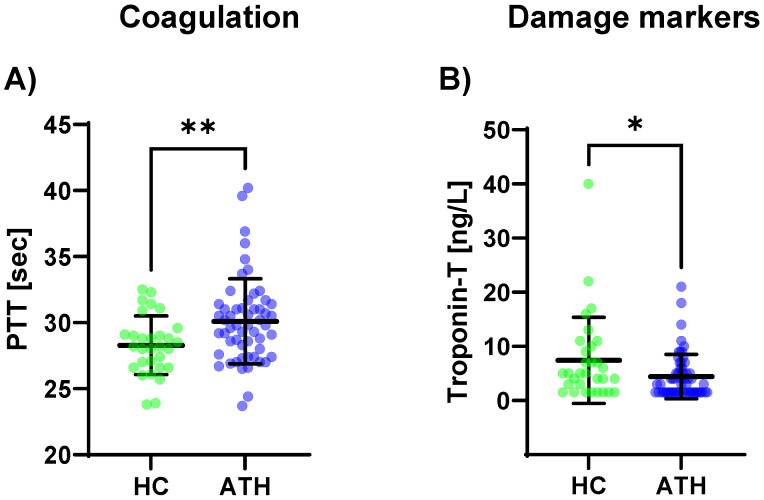
Differences in the variables grouped for (**A**) coagulation, and (**B**) damage markers between the previously COVID-19-infected athletes (ATH) and the healthy controls (HC) with comparable fitness statuses. PTT (HC 28.3 ± 2.2 s vs. ATH 30.1 ± 3.2 s) was significantly different between the ATH and HC groups. Troponin-T concentrations in the ATH group (4.4 ± 4.1 ng/L) were significantly decreased compared to the HC group (7.4 ± 8.0 ng/L), whereas there were no differences for the other seven variables grouped as damage markers (e.g., creatine kinase, urea, and NT-Pro BNP). Of the six variables grouped under coagulation, * *p* ≤ 0.05, ** *p* ≤ 0.01.

The PTT levels (*p* = 0.008) were increased in the ATH group compared to the HC group (Figure 3c). A trend was observable where there were lower D-dimer levels in the ATH group (*p* = 0.054).

#### 3.1.4. Damage Markers

Of the eight variables grouped in the cardiac/muscular damage markers, only troponin-T was decreased in the ATH group compared to the HC group (*p* = 0.034) (Figure 3B).

#### 3.1.5. Electrolytes/Micronutrients

**Figure 4:**
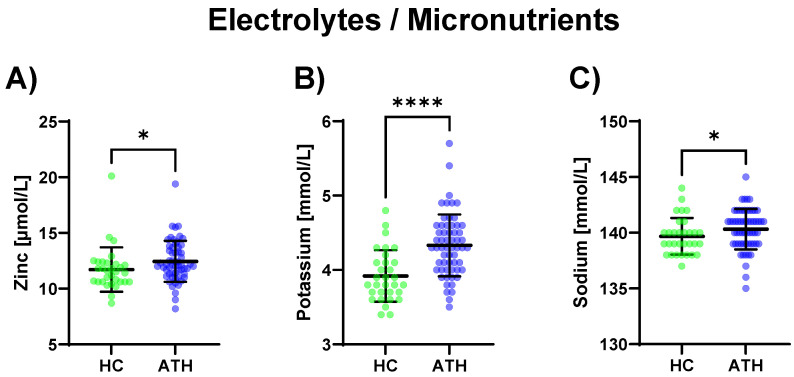
Differences in the variables grouped under electrolytes/micronutrients between the previously COVID-19-infected athletes (ATH) and the healthy controls (HC) with comparable fitness statuses. Of the five variables grouped under electrolytes/micronutrients, (**A**) zinc (HC 11.7 ± 2.0 vs. ATH 12.4 ± 1.8 μmol/L), (**B**) potassium (HC 3.9 ± 0.4 vs. ATH 4.3 ± 0.4 mmol/L), and (**C**) sodium (HC 139.7 ± 1.6 vs. ATH 140.3 ± 1.8 mmol/L) concentrations were significantly different between the ATH and HC groups. * *p* ≤ 0.05 and **** *p* ≤ 0.0001 for the ATH group compared to the HC group.

Regarding electrolytes and micronutrients, potassium (*p* < 0.0001) and sodium (*p* = 0.039), as well as zinc (*p* = 0.015), were increased in the ATH group compared to the HC group (Figure 4).

#### 3.1.6. Metabolism

**Figure 5:**
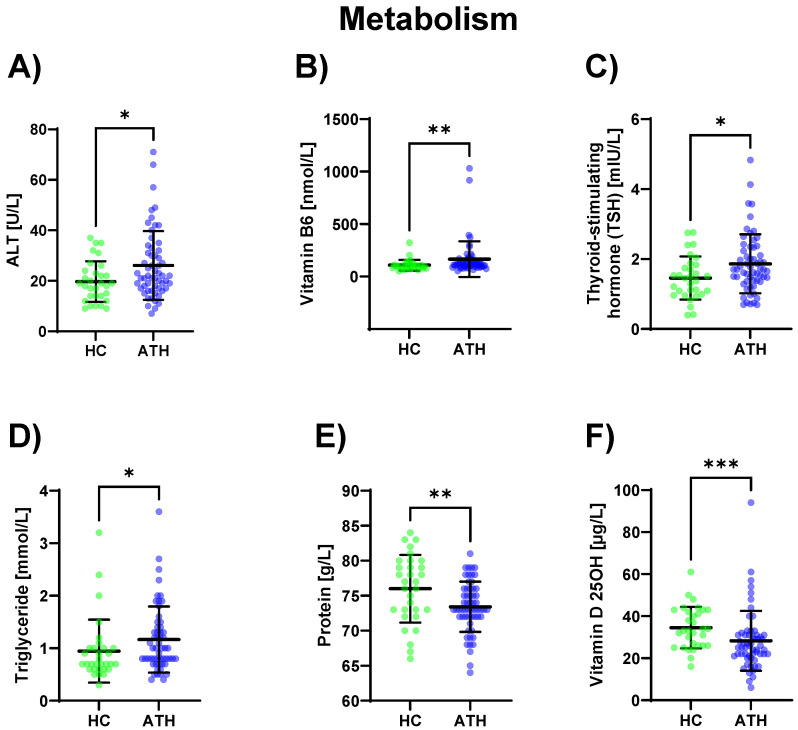
Differences in the variables grouped under metabolism between the previously COVID-19-infected athletes (ATH) and the healthy controls (HC) with comparable fitness sta-tuses. Of the elven variables grouped under metabolism, (**A**) ALT (HC 19.7 ± 8.1 vs. ATH 26.1 ± 13.6 U/L), (**B**) vitamin B6 (HC 108.4 ± 51.2 vs. ATH 165.3 ± 169.7 nmol/L), (**C**) TSH (HC 1.5 ± 0.6 vs. ATH 1.9 ± 0.8 mlU/L), (**D**) Triglyceride (HC 1.0 ± 0.6 vs. ATH 1.2 ± 0.6 mmol/L), (**E**) Protein (HC 76.0 ± 4.8 vs. ATH 73.4 ± 3.6 g/L) and (**F**) Vitamin D 25OH (HC 34.6 ± 9.9 vs. ATH 28.3 ± 14.2 μg/L) concentrations were significantly different between the ATH and HC groups. * *p* ≤ 0.05, ** *p* ≤ 0.01 and *** *p* ≤ 0.001 for the ATH group compared to the HC group.

In the ATH group, ALT (*p* = 0.031) was increased and its coenzyme vitamin B6 (*p* = 0.005) was increased compared to the HC group. Triglyceride (*p* = 0.022) concentrations were also increased in the ATH group compared to the HC group as well as the thyroid-stimulating metabolic hormone TSH (which stimulates the thyroid gland) (*p* = 0.026). Vitamin D 25OH (an antioxidant with immunostabilizing, calcium homoeostasis, bone metabolism, and muscle functions; *p* = 0.001), as well as the total protein content (*p* = 0.009), were lower in the ATH group. (Figure 5).

A detailed grouping and the results of the analyses of all variables can be found in Table S1.

### 3.2. Blood Variables: Changes in the Variables Depending on Sex

A subgroup analysis of the women between the ATH and HC groups revealed differences in the Hb content reticulocytes (*p* = 0.034), leukocyte (*p* = 0.024), soluble transferrin receptor (*p* < 0.001), absolute lymphocyte (*p* = 0.026), MCH (*p* = 0.045), MCHC (*p* = 0.021), MTV (*p* = 0.046), Neutrophile absolute (*p* = 0.035), SARS-CoV-2 Spike Antibody (*p* = 0.002), CRP (*p* = 0.038), FT3 (*p* = 0.033), FT4 (*p* = 0.015), IgE (*p* = 0.044), complement C3c (*p* = 0.009), TNF-α (*p* = 0.021), LBP (*p* = 0.002), D-dimer (*p* = 0.015), PTT (*p* = 0.023), calculated GFR CKD EPI (*p* = 0.023), potassium (*p* < 0.001), sodium (*p* = 0.032), vitamin B6 (*p* = 0.011), vitamin D25OH (*p* = 0.016), total protein content (*p* = 0.022) and thyroid-stimulating hormone (TSH) (*p* = 0.005) concentrations.

A subgroup analysis of the men between the ATH and HC groups showed significant differences in the soluble transferrin receptor (TSH) (*p* = 0.018), uric acid (*p* = 0.019), troponin-T (*p* = 0.003), potassium (*p* = 0.020), and vitamin D25OH (*p* = 0.028) concentrations.

A detailed analysis of the results of all the variables is provided in Table 1 and Table 2 of S2.

### 3.3. Blood Variables: Changes in the Variables Depending on the Time since Infection

Testing on the contribution of the variable time since infection was performed, and significant differences were observed for the ALT (*p* = 0.05), AST (*p* = 0.02), erythropoietin (*p* = 0.01), Reticulocyte absolute (*p* = 0.03), folic acid (*p* = 0.05), and TNF-alpha (*p* = 0.02) concentrations in the ATH group.

A detailed analysis of the results of all the variables is provided in Table S3.

## 4. Discussion

In this exploratory pilot analysis, blood profiles of previously COVID-19-infected professional and recreational athletes (ATH) of both sexes were compared to a healthy control group of comparable training statuses (HC). HC maintained their training volume in the weeks before the examination, which was not possible for several ATH individuals due to a prolonged recovery period after the disease. In general, ATH were not vaccinated against COVID-19, whereas HC were vaccinated.

Although there are manifold variations in symptoms and their respective severity during and after a SARS-CoV-2 infection [2], recent reviews have estimated that negative long-term health effects affect up to 80% of all COVID-19-infected people, with symptoms persisting for 4 weeks and longer [3,24].

However, it is not only individuals with comorbidities or previous disease burdens that have been hospitalized as even elite athletes with high levels of physical fitness can suffer from the effects of infection. Martinez et al. [25] screened 789 professional athletes that had tested positive for SARS-CoV-2 before returning to their sports. In total, 30 athletes had abnormal screenings (elevated troponin, abnormal ECG, and abnormal echocardiography) [25]. As athletes normally exhibit cardiovascular health with normal BMIs and less life-style associated disease burdens than the so-called general population [13,14,26], the transferability of “abnormal values” in this underrepresented group remains questionable.

After the blood profile analysis, we observed a presumably still-increased immune response in the ATH group, which was underlined by the decreased total leucocyte and the lower number of SARS-CoV-2 spike antibodies, as well as the lower CRP and LPB concentrations, compared to the HC group. In contrast to the study’s finding, an altered and persistent immune response following COVID-19 infection has been observed in different studies, with a high prevalence of increased circulating immune cells in individuals suffering from long COVID [27,28,29]. We did not test for immune cell subpopulations in our exploratory study, and so no conclusions could be drawn for, e.g., altered T-cell or B-cell activities in the ATH group, but the thresholds for leucocyte and lymphocyte concentrations in athletes may not be generally applicable due to acute [30] and/or long-term training changes, because aerobic training leads to an altered immune system reaction [31]. CRP and LBP as acute-phase proteins were decreased in the ATH group compared to the HC group. Increased CRP may be related to higher training load, and also LBP, as marker of leaky gut, may increase after training [32,33]. The moderately elevated levels in HC may be explained by high intensity exercise or competition, which can cause high CRP levels above the reference range [34,35]. HC individuals may exercise without persistent symptoms, allowing them to engage in more intense training during the period of blood sampling.

A published work by Phetsouphanh et al. [36] examined 28 especially pro- and anti-inflammatory and immunologic serum biomarkers in 31 long COVID patients and compared them with age-matched, previously SARS-CoV-2-infected but asymptomatic individuals as well as 16 healthy controls at three different time points up to 8 months after infection. They identified six biomarkers associated with long COVID and proposed a panel of these pro-inflammatory cytokines (interferon β (IFN-β), IFN-λ1, IFN-γ, CXCL9, CXCL10, IL-8, and soluble T cell immunoglobulin mucin domain) [36]. In contrast, in our study, the ATH group’s pro- or anti-inflammatory cytokine concentrations did not differ from those of the HC group. Instead, the immune systems of the ATH groups showed lower complement activations (CH50 and complement C3c levels), as well as the lower IgG levels. This is in line with the results of the decreased inflammation markers and decreased leucocytes compared to the HC group which were found in this study.

The activation of the complement system represents a fast and efficient response of the innate immune system to pathogenic invasion and is a reasonable immune adaptation to the SARS-CoV2 infection in healthy individuals [37]. In the long-term, however, the immune response might be weakened in ATH, sup-ported by the lower concentrations of IgG-antibodies and lower SARS-CoV-2 antibodies in the ATH group compared to the SARS-CoV-2-uninfected but vaccinated HC group. A pediatric study showed that children who repeatedly contracted the same disease had lower antigen-specific immunoglobulin G concentrations [38], although a different adaptation in active adult individuals should be kept in mind. This inability for a sufficient long-term immune response may increase the risk for further infections leading to persistent or recurrent exercise failure. It is known that consistent moderate exercise can improve innate immune function and has beneficial effects on the body’s defense against pathogens [39], and so perhaps long-term adaptations of the innate immune system are reasonable effects of the continuous training of the HC group. We previously showed that athletes’ immune responses differ in response to training and competition compared to those of a normal population, and an immunological matrix appears to be advisable for monitoring immune status [40].

However, there are also non-cytokine biomarkers, for example D-dimers that is also elevated in acutely infected COVID-19 patients compared to non-COVID-19-infected individuals [17]. However, this elevation seems to persist for a longer period of time. Mandal et al. [18] observed increased D-dimers in individuals suffering from long COVID [18]. In contrast, in our study, a trend for decreased D-dimers could be observed in the ATH group, assuming a reversed effect compared to the study by Mandal et al. [18]. In that study, our respective colleagues examined a study population with a mean age of 59.9 years compared with the mean age of the ATH group of 34.5 years, which showed differences in physical fitness status and age distribution compared with our study population.

Physical exercise is known to decrease iron, magnesium, and phosphorus concen-trations in erythrocytes, which may have a detrimental effect on performance [26]. The observed increase in vitamin B6, zinc, potassium, and sodium in the ATH group might be a further indication that the turnover of these micronutrients is reduced during a period of lower training volume when symptoms persist. However, this could also be influenced by dietary habits, as it can be assumed that individuals who stop exercising do not immediately change their diet. Consequently, an excess of micronutrients could be a possible result. These results of a health-oriented diet are consistent with the fact that a micronutrient deficiency is often observed in the general populations of developing and even industrialized countries with potential health and socioeconomic consequences [41,42] and that the athletic fitness also depends on sufficient macro- and micro-nutrient concentrations.

In addition, hypoproteinemia was observed during infection, and an association between COVID-19 infection and a hypercatabolic state was shown [6]. We also observed lower protein contents and vitamin D concentrations in the ATH group compared to the HC group, which confirms that the hypercatabolic state persists. A possible explanation for the hypercatabolic state is the persistence of symptoms in some patients and the following physical inactivity. Unfortunately, we did not have data on the athletes’ nutrition/supplementation behaviors during and after infection, which may have provided further insights into the different eating habits and training distributions. However, the observed data underline that regular exercise as prevention and/or adjuvant therapy may—outside of COVID-19 infection—exert positive effects on immune system function, and thus, it can presumably lower virus-induced complications [43,44].

Given the beneficial effects of regular exercise and high levels of cardiovascular fitness, together with increasing evidence for lowered COVID-19 severity and mortality in athletes [44,45], more focus on COVID-19 treatments should pertain to preventive measures such as physical exercise and maintaining a normal body weight and be prescribed and advised by all health care practitioners. As we observed in the sex-divided analyses, more differences were observed between the ATH and HC groups for the women (25 different variables, for example: absolute lymphocyte, MCH, TNF-alpha, potassium, and vitamin B6) than for the men (five different variables: TSH, uric acid, troponin-T, potassium and vitamin D 25OH), and sex-specific differences should always be kept in mind. Now, increasing numbers of studies have shown greater long-term symptom susceptibility in women after COVID-19 infection [46], and explanatory approaches have included differences in immune responses and cardiovascular comorbidities, as well as androgenic responses [47,48]. Moreover, female athletes have additional micronutritional needs and hormonal differences compared to men and their peers in the general population, and these can affect their health and performance [49]. Thus, the focus of further research and treatment decisions should always include sex-specific differences.

### Strengths and Limitations

Based on the 74 different blood variables analyzed in the athletes, a first database containing information about the long-term effects of COVID-19 in physically active individuals was obtained. Due to the comparable control group, which may also have had abnormal variable values due to physical activity, small changes in the immune and blood systems of the previously infected athletes could be detected. However, the training volumes differed slightly but not significant between the study and control groups. These small differences may have influenced the blood variables to a small extent. Moreover, the recruited study population included athletes of different sport types with a relatively heterogeneous distribution and exercise experience. In addition, this exploratory pilot was performed using the blood profiles of only 90 participants, which have may contributed to non-significant statistical results when we considered multiple testing. Attention should also be paid to the implementation period. The data were collected from March 2020 to July 2021, when the SARS-CoV-2 Alpha to Delta variants were the most prevalent. Therefore, the results are not directly applicable to populations infected with other virus variants. In addition, the effect of vaccination prior to infection may have had an impact on the changes in the blood variables. Nevertheless, further evaluation is ongoing, and this exploratory design has provided first insights into post-COVID-infection status in an up-to-now underrepresented athletic population.

## 5. Conclusions

We compared the blood profiles of athletes with and without persistent symptoms post COVID-19 with previously uninfected but vaccinated individuals. Previously SARS-CoV-2-infected athletes had lower C3c complement and lower IgG, lower Vitamin-D levels and increased electrolyte/micronutrient concentrations. The observed values may be interpreted as a different immune response in such athletes. In conclusion, more differences in immune response were found between ATH and HC in female athletes than in men. Several differences in the blood markers were related to the exercise restriction after COVID-19-infection in ATH. As this study did not aim to identify so-called post-COVID conditions or long COVID disease states, the determined values may be helpful in further examinations of such patients and in hypothesis-building for further research.

## Figures and Tables

**Table 1:** Anthropometric data, training volume before disease, symptoms during acute phase, and persistent symptoms of the study population.

		ATH		HC
Sex (f/m)		29/30		19/12
		Mean (±SD)		Mean (±SD)
Age (years)	N (59)	34.5 ± 12.2	N (31)	31.9 ± 10.4
Body mass (kg)	N (59)	73.9 ± 15.2	N (18)	72.8 ± 13.9
Height (cm)	N (59)	175.6 ± 9.3	N (21)	176.1 ± 8.93
BMI (height/(body mass)^2^)	N (59)	23.9 ± 3.9	N (18)	23.4 ± 3.5
Time since infection (months)	N (59)	3.8 ± 2.68		
Sport type *		Frequency		Frequency
Endurance		42 (60.0%)		12 (29.3%)
Resistance		11 (15.7%)		11 (26.8%)
Team or combat sports		16 (22.9%)		3 (7.3)%
Technical sports		1 (1.4%)		0
Missing		0		15 (36.6%)
Training volume (before infection)		Number		Number
0–3 h/week		0		2 (6.5%)
3–5 h/week		28 (47.5%)		6 (19.4%)
6–10 h/week		16 (27.1%)		7 (22.6%)
10–15 h/week		8 (13.6%)		0
>15 h/week		5 (8.5%)		1 (3.2%)
Missing		2 (3.4%)		15 (48.4%)
Symptoms during acute phase *		Frequency		Frequency
Fever (missing)		25 (7)		
Cough (missing)		30 (7)		
Ageusia/anosmia (missing)		16 (22)		
Rhinitis (missing)		28 (7)		
Throat pain (missing)		25 (7)		
Dyspnea under load (missing)		24 (7)		
Dyspnea during rest (missing)		18 (7)		
Diarrhea (missing)		7 (22)		
Headache (missing)		19 (22)		
Persistent symptoms *		Frequency		Frequency
Fatigue and performance decreases		33		
Sleeping disorders		15		
Neurocognitive disorders		15		
Respiratory disorders		20		
Autonomic disorders		12		
Muscle pain		11		
Psychological-related items		2		
Immunological disorders		3		
No symptoms		21		
Missing		2		

* multiple choice possible.

## Data Availability

All data are available on reasonable request from the authors. The results will be publicly available at the German Clinical Trials Register (https://www.drks.de/drks_web/navigate.do?navigationId=start, accessed on 15 July 2022) at the end of the Cosmo-S study.

## Supplementary Materials

The following supporting information can be downloaded at: https://www.mdpi.com/article/10.3390/biomedicines11071911/s1, Table S1: Differences between the ATH and HC groups regarding blood values; Table 1 and Table 2 S2: Differences between the women and men in the ATH and HC groups; and Table 1 S3: Influence of time since infection on blood variables in ATH group.

## References

[1] National Institute for Health and Care Excellence (Great Britain) (2020). COVID-19 Rapid Guideline: Managing the Long-Term Effects of COVID-19.

[2] Cirulli E.T., Schiabor Barrett K.M., Riffle S., Bolze A., Neveux I., Dabe S., Grzymski J.J., Lu J.T., Washington N.L. (2020). Long-term COVID-19 symptoms in a large unselected population, 2020. medRxiv.

[3] Lopez-Leon S., Wegman-Ostrosky T., Perelman C., Sepulveda R., Rebolledo P.A., Cuapio A., Villapol S. (2021). More than 50 Long-term effects of COVID-19: A systematic review and meta-analysis. Sci. Rep..

[4] Bizjak D.A., John L., Matits L., Uhl A., Schulz S.V.W., Schellenberg J., Peifer J., Bloch W., Weiß M., Grüner B. (2022). SARS-CoV-2 Altered Hemorheological and Hematological Parameters during One-Month Observation Period in Critically Ill COVID-19 Patients. Int. J. Mol. Sci..

[5] Barkas F., Filippas-Ntekouan S., Kosmidou M., Liberopoulos E., Liontos A., Milionis H. (2021). Anakinra in Hospitalized non-Intubated Patients with Coronavirus Disease 2019: A Systematic review and meta-analysis. Rheumatology.

[6] Al Maqbali M., Al Badi K., Al Sinani M., Madkhali N., Dickens G.L. (2022). Clinical Features of COVID-19 Patients in the First Year of Pandemic: A Systematic Review and Meta-Analysis. Biol. Res. Nurs..

[7] García L.F. (2020). Immune Response, Inflammation, and the Clinical Spectrum of COVID-19. Front. Immunol..

[8] Sudre C.H., Murray B., Varsavsky T., Graham M.S., Penfold R.S., Bowyer R.C., Pujol J.C., Klaser K., Antonelli M., Canas L.S. (2021). Attributes and predictors of long COVID. Nat. Med..

[9] Zorn J., Vollrath S., Matits L., Schönfelder M., Schulz S.V.W., Jerg A., Steinacker J.M., Bizjak D.A. (2023). Relationship between physical performance and perception of stress and recovery in daily life post COVID-19-An explorative study. PLoS ONE.

[10] Matits L., Munk M., Bizjak D.A., Kolassa I.-T., Karrasch S., Vollrath S., Jerg A., Steinacker J.M. (2023). Inflammation and severity of depressive symptoms in physically active individuals after COVID-19—An exploratory immunopsychological study investigating the effect of inflammation on depressive symptom severity. Brain Behav. Immun. Health.

[11] Kallen V., Scherder R., Cramer M.J., Stam J., Johnson B., Scherder E. (2021). Neutralizing a Springboard for Inflammation: Physical Activity to Control the Immune Network. Healthcare.

[12] Marques A., Henriques-Neto D., Peralta M., Martins J., Demetriou Y., Schönbach D.M.I., de Matos M.G. (2020). Prevalence of Physical Activity among Adolescents from 105 Low, Middle, and High-income Countries. Int. J. Environ. Res. Public Health.

[13] Banfi G., Mauri C., Morelli B., Di Gaetano N., Malgeri U., Melegati G. (2006). Reticulocyte count, mean reticulocyte volume, immature reticulocyte fraction, and mean sphered cell volume in elite athletes: Reference values and comparison with the general population. Clin. Chem. Lab. Med..

[14] Díaz Martínez A.E., Alcaide Martín M.J., González-Gross M. (2022). Basal Values of Biochemical and Hematological Parameters in Elite Athletes. Int. J. Environ. Res. Public Health.

[15] Pedlar C.R., Newell J., Lewis N.A. (2019). Blood Biomarker Profiling and Monitoring for High-Performance Physiology and Nutrition: Current Perspectives, Limitations and Recommendations. Sports Med..

[16] Del Valle D.M., Kim-Schulze S., Huang H.-H., Beckmann N.D., Nirenberg S., Wang B., Lavin Y., Swartz T.H., Madduri D., Stock A. (2020). An inflammatory cytokine signature predicts COVID-19 severity and survival. Nat. Med..

[17] Leisman D.E., Ronner L., Pinotti R., Taylor M.D., Sinha P., Calfee C.S., Hirayama A.V., Mastroini F., Turtle C.J., Harhay M.O. (2020). Cytokine elevation in severe and critical COVID-19: A rapid systematic review, meta-analysis, and comparison with inflammatroy syndromes. Lancet.

[18] Mandal S., Barnett J., Brill S.E., Brown J.S., Denneny E.K., Hare S.S., Heightman M., Hillman T.E., Jacob J., Jarvis H.C. (2021). ‘Long-COVID’: A cross-sectional study of persisting symptoms, biomarker and imaging abnormalities following hospitalisation for COVID-19. Thorax.

[19] Grazioli S., Tavaglione F., Torriani G., Wagner N., Rohr M., L’Huillier A.G., Leclercq C., Perrin A., Bordessoule A., Beghetti M. (2021). Immunological Assessment of Pediatric Multisystem Inflammatory Syndrome Related to Coronavirus Disease 2019. J. Pediatr. Infect. Dis. Soc..

[20] Conti P., Ronconi G., Caraffa A., Gallenga C.E., Ross R., Frydas I., Kritas S.K. (2020). Induction of pro-inflammatory cytokines (IL-1 and IL-6 and lung inflammation by coronavirus-19 (Covi-19 or SARS-CoV-2): Anti-inflammatory strategies. J. Biol. Regul. Homeost. Agents.

[21] Niess A.M., Widmann M., Gaidai R., Gölz C., Schubert I., Castillo K., Sachs J.P., Bizjak D., Vollrath S., Wimbauer F. (2022). COVID-19 in German Competitive Sports: Protocol for a Prospective Multicenter Cohort Study (CoSmo-S). Int. J. Public Health.

[22] Jetté M., Sidney K., Blümchen G. (1990). Metabolic equivalents (METS) in exercise testing, exercise prescription, and evaluation of functional capacity. Clin. Cardiol..

[23] Wang Z. Unified Robust Estimation, 2020. https://arxiv.org/pdf/2010.02848.

[24] Domingo F.R., Waddell L.A., Cheung A.M., Cooper C.L., Belcourt V.J., Zuckermann A.M.E., Corrin T., Ahmad R., Boland L., Laprise C. (2021). Prevalence of long-term effects in individuals diagnosed with COVID-19: An updated living systematic review. MedRxiv.

[25] Martinez M.W., Tucker A.M., Bloom O.J., Green G., DiFiori J.P., Solomon G., Phelan D., Kim J.H., Meeuwisse W., Sills A.K. (2021). Prevalence of Inflammatory Heart Disease Among Professional Athletes with Prior COVID-19 Infection Who Received Systematic Return-to-Play Cardiac Screening. JAMA Cardiol..

[26] Mariño M.M., Grijota F.J., Bartolomé I., Siquier-Coll J., Román V.T., Muñoz D. (2020). Influence of physical training on erythrocyte concentrations of iron, phosphorus and magnesium. J. Int. Soc. Sports Nutr..

[27] Galán M., Vigón L., Fuertes D., Murciano-Antón M.A., Casado-Fernández G., Domínguez-Mateos S., Mateos E., Ramos-Martín F., Planelles V., Torres M. (2022). Persistent Overactive Cytotoxic Immune Response in a Spanish Cohort of Individuals With Long-COVID: Identification of Diagnostic Biomarkers. Front. Immunol..

[28] Glynne P., Tahmasebi N., Gant V., Gupta R. (2022). Long COVID following mild SARS-CoV-2 infection: Characteristic T cell alterations and response to antihistamines. J. Investig. Med..

[29] Siemińska I., Węglarczyk K., Surmiak M., Kurowska-Baran D., Sanak M., Siedlar M., Baran J. (2021). Mild and Asymptomatic COVID-19 Convalescents Present Long-Term Endotype of Immunosuppression Associated With Neutrophil Subsets Possessing Regulatory Functions. Front. Immunol..

[30] Gomes J.H., Mendes R.R., Franca C.S., Da Silva-Grigoletto M.E., Da Pereira Silva D.R., Antoniolli A.R., de Oliveira e Silva A.M., Quintans-Júnior L.J. (2020). Acute leucocyte, muscle damage, and stress marker responses to high-intensity functional training. PLoS ONE.

[31] Balogh L., Szabó K., Pucsok J.M., Jámbor I., Gyetvai Á., Mile M., Barna L., Szodoray P., Tarr T., Csiki Z. (2022). The Effect of Aerobic Exercise and Low-Impact Pilates Workout on the Adaptive Immune System. J. Clin. Med..

[32] Wallett A., Périard J.D., Saunders P., McKune A. (2021). Effect of Exercising in the Heat on Intestinal Fatty Acid-Binding Protein, Endotoxins, and Lipopolysaccharide-Binding Protein Markers in Trained Athletic Populations: A Systematic Literature Review. Int. J. Sport Nutr. Exerc. Metab..

[33] Kavanagh K., Hsu F.-C., Davis A.T., Kritchevsky S.B., Rejeski W.J., Kim S. (2019). Biomarkers of leaky gut are related to in-flammation and reduced physical function in older adults with cardiometabolic disease and mobility limitations. Geroscience.

[34] Kleiven Ø., Bjørkavoll-Bergseth M., Melberg T., Skadberg Ø., Bergseth R., Selvåg J., Auestad B., Aukrust P., Aarsland T., Ørn S. (2018). High physical fitness is associated with reduction in basal- and exercise-induced inflammation. Scand. J. Med. Sci. Sports.

[35] Nyborg C., Melau J., Bonnevie-Svendsen M., Mathiasen M., Melsom H.S., Storsve A.B., Hisdal J. (2020). Biochemical markers after the Norseman Extreme Triathlon. PLoS ONE.

[36] Phetsouphanh C., Darley D.R., Wilson D.B., Howe A., Munier C.M.L., Patel S.K., Juno J.A., Burrell L.M., Kent S.J., Dore G.J. (2022). Immunological dysfunction persists for 8 months following initial mild-to-moderate SARS-CoV-2 infection. Nat. Immunol..

[37] Hollenberg M.D., Epstein M. (2022). The innate immune response, microenvironment proteinases, and the COVID-19 pandemic: Pathophysiologic mechanisms and emerging therapeutic targets. Kidney Int. Suppl..

[38] Sharma S.K., Casey J.R., Pichichero M.E. (2012). Reduced serum IgG responses to pneumococcal antigens in otitis-prone children may be due to poor memory B-cell generation. J. Infect. Dis..

[39] Shephard R.J., Shek P.N. (1994). Potential impact of physical activity and sport on the immune system—a brief review. Br. J. Sports Med..

[40] Bizjak D.A., Treff G., Zügel M., Schumann U., Winkert K., Schneider M., Abendroth D., Steinacker J.M. (2021). Differences in Immune Response During Competition and Preparation Phase in Elite Rowers. Front. Physiol..

[41] Rautiainen S., Manson J.E., Lichtenstein A.H., Sesso H.D. (2016). Dietary supplements and disease prevention—A global overview. Nat. Rev. Endocrinol..

[42] Bailey R.L., West K.P., Black R.E. (2015). The epidemiology of global micronutrient deficiencies. Ann. Nutr. Metab..

[43] Yisak H., Ewunetei A., Kefale B., Mamuye M., Teshome F., Ambaw B., Yideg Yitbarek G. (2021). Effects of Vitamin D on COVID-19 Infection and Prognosis: A Systematic Review. Risk Manag. Healthc. Policy.

[44] Nieman D.C. (2021). Exercise Is Medicine for Immune Function: Implication for COVID-19. Curr. Sports Med. Rep..

[45] Jakobsson J., Cotgreave I., Furberg M., Arnberg N., Svensson M. (2021). Potential Physiological and Cellular Mechanisms of Exercise That Decrease the Risk of Severe Complications and Mortality Following SARS-CoV-2 Infection. Sports.

[46] Bai F., Tomasoni D., Falcinella C., Barbanotti D., Castoldi R., Mulè G., Augello M., Mondatore D., Allegrini M., Cona A. (2022). Female gender is associated with long COVID syndrome: A prospective cohort study. Clin. Microbiol. Infect..

[47] Bienvenu L.A., Noonan J., Wang X., Peter K. (2020). Higher mortality of COVID-19 in males: Sex differences in immune response and cardiovascular comorbidities. Cardiovasc. Res..

[48] Mohamed M.S., Moulin T.C., Schiöth H.B. (2021). Sex differences in COVID-19: The role of androgens in disease severity and progression. Endocrine.

[49] Holtzman B., Ackerman K.E. (2021). Recommendations and Nutritional Considerations for Female Athletes: Health and Performance. Sports Med..

