# Supplementary material for: Blood Profiling of Athletes after COVID-19: Differences in Blood Profiles of Post-COVID-19 Athletes Compared to Uninfected Athletic Individuals—An Exploratory Analysis
Source: Biomedicines. 2023 Jul 6;11(7):1911. doi: 10.3390/biomedicines11071911 (PMC10377547; doi:10.3390/biomedicines11071911)
Supplement: Supplementary file 1 [file biomedicines-11-01911-s001.zip › Table S1.pdf]

Table S1

Differences between ATH and HC regarding blood values

	**ATH (n = 59)**			**HC (n = 31)**					
	mean	SD		mean	SD	$W^{\#}$	*p*	*d*	[95% CI]
**Blood cell (count)**									
Basophile absolute [10^9/L]	0.04	0.01		0.04	0.02	1057.5	0.214	0.20	[-0.24; 0.64]
Basophile relative [%]	0.67	0.22		0.66	0.29	999.0	0.471	0.05	[-0.39; 0.48]
Eosinophile absolute [10^9/L]	0.13	0.10		0.12	0.12	1078.0	0.165	0.13	[-0.30; 0.57]
Eosinophile relative [%]	2.14	1.70		2.02	1.62	952.5	0.750	0.08	[-0.36; 0.51]
Erythropoietin [mIU/ml]	11.37	3.87		11.05	4.37	955.0	0.535	0.08	[-0.36; 0.51]
Red blood cell distribution width (RDW) [%]	12.55	0.69		12.58	0.59	843.5	0.549	-0.04	[-0.48; 0.39]
Erythrocyte [10^{12}/L]	4.61	0.50		4.74	0.44	774.0	0.233	-0.29	[-0.73; 0.15]
Ferritin [µg/L]	92.97	77.57		109.86	95.72	840.5	0.532	-0.19	[-0.62; 0.25]
Hb content reticulocytes [pg]	33.92	1.47		33.36	1.05	1137.5	0.059	0.47	[0.03; 0.91]
Hematocrit [%]	0.41	0.03		0.42	0.03	844.0	0.551	-0.15	[-0.58; 0.29]
Hemoglobin [g/dL]	13.96	1.12		14.06	1.26	888.5	0.828	-0.08	[-0.52; 0.35]
**Leukocyte [10^9/L]**	6.34	1.02		5.89	1.47	1164.5	**0.034**	0.33	[-0.10; 0.77]

**Soluble Transferrin receptor [mg/L]**	4.26	1.72		2.83	0.61	1448	**<0.001**	1.28	[0.80; 1.75]
**Lymphocyte absolute [10^9/L]**	1.80	0.46		2.01	0.38	1181.5	**0.023**	0.49	[0.05; 0.93]
Lymphocyte relative [%]	32.08	6.31		31.07	7.69	943.0	0.812	0.14	[-0.30; 0.57]
MCH [pg]	30.33	1.43		29.75	0.84	1131.5	0.066	0.53	[0.09; 0.97]
MCHC [g/dL]	33.67	1.02		33.86	0.78	1073	0.179	0.20	[-0.24; 0.64]
MCV [fl]	89.60	4.50		88.43	3.10	1054.5	0.236	0.32	[-0.12; 0.76]
Monocyte absolute [10^9/L]	0.50	0.16		0.48	0.15	968.0	0.644	0.11	[-0.32; 0.55]
Monocyte relative [%]	7.76	2.40		8.13	1.95	788.5	0.286	-0.17	[-0.61; 0.26]
MTV [fl]	10.46	1.24		10.75	1.14	772.0	0.228	-0.25	[-0.69; 0.18]
Neutrophile absolute [10^9/L]	3.66	0.89		3.48	1.32	1128.0	0.070	0.15	[-0.29; 0.59]
Neutrophile relative [%]	57.36	7.92		58.13	8.72	916.0	0.993	-0.09	[-0.53; 0.34]
Reticulocyte absolute [10^9/L]	59.38	20.08		62.59	15.38	1034.5	0.310	0.17	[-0.26; 0.61]
Reticulocyte relative [%]	1.37	0.35		1.25	0.38	1103.5	0.109	0.32	[-0.12; 0.76]
Immature Reticulocytes [%]	7.37	3.70		7.14	2.53	898.5	0.895	0.08	[-0.36; 0.51
Thrombocyte [10^9/L]	255.97	52.20		249.66	47.31	999.0	0.476	0.13	[-0.31; 0.56]
**Inflammation/Immunology**									
**SARS-CoV2 Spike Antibody [U/mL]**	2156.84	2324.28		597.56	564.44	408.0	**<0.001**	0.81	[0.18;1.43]

**CH50 [2] [U/mL]**	54.57	4.44		50.42	5.94	249.0	**0.045**	0.73	[0.01;1.45]
**C-Reactive Protein [1] [mg/L]**	1.29	2.23		0.63	0.60	1181.0	**0.012**	0.48	[0.04; 0.92]
FT3 [pmol/L]	5.05	0.80		4.90	0.79	1071.5	0.139	0.19	[-0.25; 0.62]
FT4 [pmol/L]	16.28	2.73		15.26	2.38	1078.0	0.124	0.40	[-0.04; 0.84]
IgA [g/L]	1.71	0.65		1.89	0.90	776.5	0.486	-0.21	[-0.66; 0.23]
IgE [IU/mL]	148.34	222.52		128.20	324.72	1125.0	0.052	0.07	[-0.37; 0.50]
**IgG [g/L]**	12.00	2.72		10.48	1.63	1168.0	**0.013**	0.73	[0.2;1.18]
IgM [g/L]	1.25	0.60		1.09	0.55	1031.5	0.198	0.28	[-0.16; 0.71]
IL-1β [1] [pg/mL]	6.97	7.80		6.34	6.70	874.0	0.733	0.09	[-0.35; 0.52]
IL-10 [1] [pg/mL]	2.59	4.17		1.91	2.94	835.0	0.875	0.20	[-0.24; 0.64]
IL- 6[1] [pg/mL]	1.29	0.85		1.11	0.80	1000.5	0.279	0.23	[-0.21; 0.66]
IL-8 [1] [ng/L]	4.25	2.90		3.64	2.00	1015.5	0.317	0.26	[-0.18; 0.70]
**Complement C3c [g/L]**	1.13	0.16		1.04	0.15	903.0	**0.030**	0.55	[0.08;1.02]
Complement C4 [g/L]	0.23	0.06		0.21	0.06	847.5	0.113	0.31	[-0.15; 0.77]
TNF-α [pg/mL]	4.70	1.63		4.35	1.64	979.0	0.327	0.22	[-0.23; 0.65]
**LBP [µg/mL]**	5.04	1.42		4.12	1.06	1214.0	**0.004**	0.77	[0.31;1.22]
**Coagulation**									
D-Dimers [1] [mg/IU]	0.18	0.13		0.13	0.09	964.5	0.054	0.40	[-0.05; 0.85]

Fibrinogen [g/L]	2.72	0.34		2.59	0.40	985.5	0.141	0.36	[-0.09; 0.80]
Folic acid [nmol/L]	19.87	9.01		19.54	8.43	739.5	0.768	0.04	[-0.42; 0.50]
Thrombin time [sec]	17.06	0.59		17.06	0.79	865.0	0.716	0.00	[-0.45; 0.44]
**PTT [sec]**	28.29	2.22		30.10	3.21	537.0	**0.008**	-0.62	[-1.08; -0.17]
**Quick [%] (Internal laboratory calculation – not external comparable)**	96.66	16.52		89.73	15.17	1018.0	**0.038**	0.44	[-0.01; 0.90]
**Damage Markers**									
Calculated GFR CKD EPI [mL/min]	106.65	17.97		100.03	16.35	1124.0	0.076	0.39	[-0.05; 0.83]
CK [U/L]	309.84	668.95		165.59	134.93	858.0	0.634	0.36	[-0.08; 0.79]
Urea [mmol/L]	4.54	1.61		4.73	1.54	817.5	0.412	-0.13	[-0.56; 0.31]
Uric Acid [µmol/L]	260.42	49.34		287.24	70.33	729.0	0.116	-0.42	[-0.86; 0.02]
Lactate Dehydrogenase (LDH) [U/L]	193.87	39.49		195.95	32.36	896.0	0.878	-0.06	[-0.49; 0.38]
Myoglobin [µg/L]	76.74	199.31		35.24	17.09	423.0	0.501	0.35	[-0.17; 0.86]
NT pro BNP [pg/mL]	58.26	49.42		51.63	39.57	978.0	0.422	0.15	[-0.29; 0.59]
**Troponin-T[1] [ng/L]**	7.42	7.95		4.44	4.10	1156.5	**0.035**	0.52	[0.08; 0.96
**Electrolytes/Micronutrients**									
**Zinc [µmol/L]**	11.72	1.99		12.44	1.84	617.5	**0.015**	-0.38	[-0.82; 0.06

Glucose [mg/dL]	80.19	14.47		82.80	11.81	780.0	0.255	-0.20	[-0.64; 0.23]
**Potassium [mmol/L]**	3.92	0.35		4.33	0.42	394.0	**<0.001**	-1.04	[-1.50; -0.58]
**Sodium [mmol/L]**	139.68	1.64		140.32	1.81	675.0	**0.039**	-0.37	[-0.80; 0.07
Selen [µg/L]	77.70	15.46		77.25	16.68	883.5	0.894	0.03	[-0.41; 0.47]
**Vitamins/Metabolism**									
Vitamin B1 [nmol/L]	142.70	23.51		134.86	27.36	956.5	0.367	0.30	[-0.14; 0.74
Vitamin B12 [pmol/L]	308.65	139.47		332.18	163.02	800.5	0.643	-0.15	[-0.59; 0.29
**Vitamin B6 [nmol/L]**	108.37	51.17		165.28	169.74	539.5	**0.005**	-0.40	[-0.85; 0.04
**Vitamin D25OH [µg/L]**	34.58	9.91		28.29	14.22	1279.5	**0.001**	0.49	[0.04; 0.93
**Protein [g/L]**	76.00	4.84		73.41	3.59	1202.5	**0.009**	0.64	[0.19;1.08]
**ALT [U/L]**	19.71	8.07		26.12	13.60	648.0	**0.031**	-0.54	[-0.98; -0.09]
AST [U/L]	28.84	15.40		29.36	10.99	787.0	0.280	-0.04	[-0.48; 0.39]
**Thyroid-stimulating hormone (TSH) [mlU/L]**	1.46	0.62		1.86	0.84	651.5	**0.026**	-0.53	[-0.97; -0.08
Creatinine [µmol/L]	72.19	17.63		77.08	13.76	685.0	0.052	-0.32	[-0.76; 0.12]
**Triglyceride [mmol/L]**	0.95	0.60		1.16	0.63	610.5	**0.022**	-0.35	[-0.79; 0.09]
Cholesterol [mmol/L]	4.62	1.02		4.56	0.85	855.5	0.915	0.07	[-0.37; 0.50]

Note. All measures are given as mean and SD. Significant group effects are given in bold.

[1] Concentrations lower limit of detection were replaced by half of the detection limit (LOD): IL-1β < 1.5 pg /ml (LOD: 14), IL-6 < 1.5 pg /ml (LOD: 14), IL-8 < 2.0 pg/ ml (LOD: 12), IL-10 < 1.0 pg/ml (LOD: 31), CRP < 0.6 mg/l (LOD =51), D-dimers < 0.2 mg/l FEU (LOD: 63), Troponin-T < 3.0 ng/l (LOD: 33).

[2] 30 individuals with CH50 concentrations > 60 were excluded from the analysis of CH50. 7 individuals with folic acid concentrations > 45.4 were excluded from the analysis of folic acid.

Two-tailed Man-Whitney U- Test. Cohen's d is reported as effect size measure [95%-CI of d]
