# Supplementary material for: Blood Profiling of Athletes after COVID-19: Differences in Blood Profiles of Post-COVID-19 Athletes Compared to Uninfected Athletic Individuals—An Exploratory Analysis
Source: Biomedicines. 2023 Jul 6;11(7):1911. doi: 10.3390/biomedicines11071911 (PMC10377547; doi:10.3390/biomedicines11071911)
Supplement: Supplementary file 1 [file biomedicines-11-01911-s001.zip › Table S2.pdf]

Table S2a

Differences between ATH and HC-Women

	ATH			HC					
	Mean	SD		Mean	SD	*W*	*p*	*d*[#]	95%-CI
**Blood cell (count)**									
Basophile absolute [10^9/L]	0.04	0.01		0.04	000.01	277.0	0.719	-0.06	[-0.64;0.53]
Basophile relative [%]	0.59	0.15		0.65	000.26	234.5	0.563	-0.26	[-0.85;0.33]
Eosinophile absolute [10^9/L]	0.10	0.05		0.10	000.09	288.5	0.553	-0.06	[-0.65;0.53]
Eosinophile relative [%]	1.46	0.75		1.80	100.39	247.5	0.776	-0.29	[-0.88;0.30]
Erythropoietin [mIU/ml]	11.73	4.59		11.73	500.50	259.5	0.875	0.00	[-0.59;0.59]
Red blood cell distribution width (RDW) [%]	12.62	0.81		12.57	000.57	252.5	0.861	0.07	[-0.52;0.66]
Erythrocyte [10^{12}/L]	4.39	0.35		4.41	000.24	253.5	0.877	-0.09	[-0.67;0.50]
Ferritin [µg/L]	55.89	38.59		51.07	3700.54	281.0	0.669	0.13	[-0.46;0.72]
Hb content reticulocytes [pg]	34.01	1.48		33.13	100.22	349.5	0.054	0.66	[0.05;1.26]
Hematocrit [%]	0.40	0.02		0.39	000.02	292.5	0.493	0.16	[-0.43;0.75]

Hemoglobin [g/dL]	13.42	0.71		13.12	000.71	328.5	0.142	0.43	[-0.17;1.02]
**Leukocyte [10^9/L]**	6.54	0.74		5.91	100.58	356.0	**0.038**	0.47	[-0.13;1.07]
**Soluble Transferrin receptor [mg/L]**	4.45	1.72		2.96	000.68	420.5	**0.000**	1.26	[0.61;1.89]
**Lymphocyte absolute [10^9/L]**	1.79	0.52		2.06	000.35	355.5	**0.039**	0.58	[-0.02; 1.18]
Lymphocyte relative [%]	31.69	5.96		31.10	900.00	243.0	0.702	0.07	[-0.52;0.66]
MCH [pg]	30.54	1.44		29.79	000.81	342.0	0.078	0.70	[0.09;1.30]
**MCHC [g/dL]**	33.32	0.90		33.88	000.89	366.5	**0.021**	0.61	[0.01; 1.21]
MCV [fl]	90.18	4.42		89.44	300.08	274.0	0.784	0.20	[-0.39;0.79]
Monocyte absolute [10^9/L]	0.49	0.16		0.45	000.14	309.0	0.277	0.27	[-0.32;0.86]
Monocyte relative [%]	7.58	2.55		7.69	100.93	241.5	0.677	-0.05	[-0.64;0.54]
**MTV [fl]**	10.18	1.18		10.87	100.30	167.0	**0.041**	-0.55	[-1.14;0.06]
Neutrophile absolute [10^9/L]	3.85	0.74		3.56	100.55	350.0	0.053	0.22	[-0.37;0.81]
Neutrophile relative [%]	58.70	7.15		58.77	1000.05	284.5	0.615	-0.01	[-0.60;0.58]
Reticulocyte absolute [10^9/L]	56.95	17.25		61.98	1600.26	303	0.367	0.30	[-0.30; 0.89]
Reticulocyte relative [%]	1.42	0.38		1.29	000.37	313.0	0.26	0.35	[-0.25;0.94]

Immature Reticulocytes [%]	7.52	3.05		7.51	200.69	265.0	0.939	0.00	[-0.58;0.60]
Thrombocyte [10^9/L]	275.11	42.60		254.62	3800.71	333.0	0.118	0.51	[-0.09;1.10]
**Inflammation/Immunology**									
**SARS-CoV2 Spike Antibody [U/mL]**	2597.83	2431.28		748.00	67900.22	124.0	**0.003**	0.89	[0.01;1.75]
CH50 [2] [U/mL]	54.15	5.55		50.74	600.13	62.0	0.205	0.57	[-0.40;1.53]
C-Reactive Protein [1] [mg/L]	1.65	2.84		0.72	000.69	346.0	0.051	0.51	[-0.09;1.10]
FT3 [pmol/L]	4.96	0.53		4.69	000.91	349.0	0.055	0.35	[-0.25;0.94]
**FT4 [pmol/L]**	16.32	2.40		14.75	200.06	362.0	**0.028**	0.71	[0.10;1.32]
IgA [g/L]	1.61	0.72		1.80	000.98	209.5	0.512	-0.22	[-0.82;0.39]
IgE [IU/mL]	153.58	198.90		63.27	9100.04	349.0	0.055	0.64	[0.03;1.24]
IgG [g/L]	12.03	2.76		10.44	100.73	329.5	0.083	0.73	[0.11;1.33]
IgM [g/L]	1.40	0.65		1.22	000.56	292.5	0.368	0.29	[-0.31;0.88]
IL-1β [1] [pg/mL]	7.06	7.16		5.62	600.09	274.5	0.775	0.22	[-0.37;0.81]
IL-10 [1] [pg/mL]	2.35	3.29		1.20	000.96	258.0	0.558	0.52	[-0.10;1.12]
IL- 6[1] [pg/mL]	1.17	0.86		1.18	100.00	255.0	0.875	-0.01	[-0.60;0.58]

IL-8 [1] [ng/L]	4.69	3.56		3.71	200.04	297.5	0.430	0.36	[-0.23;0.95]
**Complement C3c [g/L]**	1.16	0.17		1.03	000.16	288.5	**0.014**	0.83	[0.17;1.47]
Complement C4 [g/L]	0.23	0.07		0.21	000.07	250.0	0.160	0.35	[-0.28;0.98]
**TNF-α [pg/mL]**	5.06	1.88		3.89	100.10	342.0	**0.022**	0.80	[0.18;1.41]
**LBP [µg/mL]**	5.34	1.61		3.94	000.99	378.5	**0.005**	1.11	[0.47;1.74]
**Coagulation**									
**D-Dimers [1] [mg/IU]**	0.18	0.10		0.12	000.07	305.0	**0.027**	0.66	[0.04;1.27]
Fibrinogen [g/L]	2.83	0.33		2.70	000.41	286.5	0.319	0.34	[-0.27;0.94]
Folic acid [nmol/L]	20.54	10.01		21.61	800.34	186.0	0.59	-0.12	[-0.73;0.50]
Thrombin time [sec]	17.10	0.59		17.19	000.83	248.0	0.917	-0.12	[-0.72;0.47]
**PTT [sec]**	27.78	2.37		29.63	300.18	143.5	**0.022**	-0.64	[-1.25;-0.03]
Quick [%](Internal laboratory calculation – not externally comparable)	103.06	14.13		93.89	1700.51	301.5	0.085	0.56	[-0.06;1.18]
**Damage Markers**									
**Calculated GFR CKD EPI [mL/min]**	110.61	14.75		98.17	1800.56	359.5	**0.032**	0.72	[0.11;1.33]
CK [U/L]	96.00	63.37		100.90	6900.17	247.5	0.776	-0.07	[-0.66;0.52]

Urea [mmol/L]	3.82	0.95		3.98	100.18	244.0	0.718	-0.14	[-0.73;0.44]
Uric Acid [µmol/L]	246.61	38.11		239.66	3700.91	287.5	0.569	0.18	[-0.41;0.77]
Lactate Dehydrogenase (LDH) [U/L]	180.00	35.34		185.17	2500.42	233.5	0.554	-0.17	[-0.76;0.42]
Myoglobin [µg/L]	25.50	4.86		30.73	900.89	44.0	0.089	-0.63	[-1.45;0.20]
NT pro BNP [pg/mL]	64.72	58.99		70.01	4500.43	220.5	0.562	-0.10	[-0.70;0.50]
Troponin-T[1] [ng/L]	3.22	1.83		2.83	200.59	321.0	0.142	0.17	[-0.42;0.76]
**Electrolytes/Micronutrients**									
Zinc [µmol/L]	11.32	1.28		11.78	100.62	204.5	0.29	-0.30	[-0.90;0.29]
Glucose [mg/dL	82.50	13.60		83.52	900.13	229.5	0.497	-0.09	[-0.68;0.50]
**Potassium [mmol/L]**	3.84	0.30		4.29	000.43	94.0	**0.000**	-1.17	[-1.80;-0.53]
**Sodium [mmol/L]**	139.00	1.14		139.72	100.81	171.0	**0.046**	-0.46	[-1.05;0.14]
Selen [µg/L]	75.54	16.75		73.99	1900.76	257.5	0.746	0.08	[-0.51;0.68]
**Vitamins/Metabolism**									
Vitamin B1 [nmol/L]	138.22	21.52		132.28	2700.41	289.0	0.547	0.23	[-0.36;0.82]
Vitamin B12 [pmol/L]	279.78	140.31		335.93	13900.09	183.5	0.172	-0.40	[-1.00;0.20]

**Vitamin B6 [nmol/L]**	102.33	34.18		167.17	17900.10	153.5	**0.019**	-0.45	[-1.05;0.14]
**Vitamin D25OH [µg/L]**	34.50	9.24		30.10	1500.47	370.5	**0.017**	0.33	[-0.27;0.92]
ALT [U/L]	15.33	4.78		20.46	900.24	173.5	0.079	-0.66	[-1.26;-0.04]
AST [U/L]	21.61	3.40		24.07	700.44	216.0	0.328	-0.39	[-0.99;0.20]
**Protein [g/L]**	75.78	4.19		72.79	300.97	355.0	**0.039**	0.74	[0.12;1.34]
**Thyroid-stimulating hormone (TSH) [mlU/L]**	1.36	0.57		1.95	000.79	136.0	**0.006**	-0.83	[-1.43;-0.21]
Creatinine [µmol/L]	63.11	9.42		69.17	1200.33	186.0	0.103	-0.54	[-1.13;0.07]
Triglyceride [mmol/L]	0.86	0.46		1.04	000.51	182.5	0.118	-0.38	[-0.98;0.22]
Cholesterol [mmol/L]	4.67	0.79		4.68	000.82	244.0	0.866	0.00	[-0.60;0.59]

Note.

[1] Concentrations lower limit of detection were replaced by half of the detection limit (LOD)

[2] 19 individuals with CH50 concentrations > 60 were excluded from the analysis of CH50. 3 individuals with folic acid concentrations > 45.4 were excluded from the analysis of folic acid.

Two-tailed Man-Whitney U- Test. Cohen's d is reported as effect size measure [95%-CI of d]

Table S2b

Differences between ATH and HC-Men

	ATH			HC					
	**Mean**	**SD**		**Mean**	**SD**	***W***	***p***	***d***#	**95%-CI**
**Blood cell (count)**									
Basophile absolute [10^9/L]	0.05	0.02		0.04	.02	249.5	.147	0.46	[-0.20;1.12]
Basophile relative [%]	0.78	0.25		0.67	.32	247.5	.167	0.39	[-0.27;1.04]
Eosinophile absolute [10^9/L]	0.18	0.14		0.14	.14	257.5	.100	0.36	[-0.30;1.01]
Eosinophile relative [%]	3.08	2.19		2.22	1.81	242.5	.213	0.45	[-0.21;1.10]
Erythropoietin [mIU/ml]	10.88	2.68		10.39	2.83	208.0	.605	0.17	[-0.48;0.83]
Red blood cell distribution width (RDW) [%]	12.45	0.49		12.58	.61	168.5	.490	-0.22	[-0.88;0.43]
Erythrocyte [10^{12}/L]	4.91	0.52		5.06	.36	176.5	.632	-0.36	[-1.01;0.30]
Ferritin [µg/L]	144.31	89.53		166.70	10.83	16.0	.366	-0.23	[-0.88;0.42]
Hb content reticulocytes [pg]	33.80	1.50		33.57	.82	215.5	.597	0.22	[-0.44;0.87]

Hematocrit [%]	0.43	0.03		0.44	.02	182.0	.739	-0.23	[-0.89;0.42]
Hemoglobin [g/dL]	14.72	1.17		14.98	.96	181.0	.721	-0.26	[-0.91;0.40]
Leukocyte [10^9/L]	6.06	1.29		5.88	1.37	221.5	.491	0.14	[-0.52;0.79]
**Soluble Transferrin receptor [mg/L]**	4.0	1.76		2.70	.52	295.5	**.000**	1.24	[0.53;1.93]
Lymphocyte absolute [10^9/L]	1.8	0.41		1.95	0.43	236	.283	0.36	[-0.30; 1.01]
Lymphocyte relative [%]	32.62	6.97		31.05	6.34	22.0	.517	0.24	[-0.41;0.89]
MCH [pg]	30.03	1.42		29.72	.88	22.0	.517	0.29	[-0.37;0.94]
MCHC [g/dL]	34.00	1.03		33.83	0.62	188.0	.863	-0.18	[-0.83, 0.47]
MCV [fl]	88.80	4.66		87.45	2.82	23.5	.354	0.39	[-0.27;1.04]
Monocyte absolute [10^9/L]	0.51	0.17		0.51	.16	189.5	.893	-0.01	[-0.67;0.64]
Monocyte relative [%]	8.02	2.26		8.55	1.90	166.5	.459	-0.27	[-0.92;0.39]
MTV [fl]	10.84	1.26		10.64	.96	214.5	.615	0.19	[-0.47;0.84]
Neutrophile absolute [10^9/L]	3.41	1.04		3.41	1.09	202.5	.853	-0.01	[-0.66;0.65]
Neutrophile relative [%]	55.51	8.84		57.51	7.33	167.5	.475	-0.26	[-0.91;0.40]
Reticulocyte absolute [10^9/L]	61.74	22.51		63.43	14.67	219.5	.526	0.08	[-0.57, 0.73]

Reticulocyte relative [%]	1.30	0.30		1.21	.38	232.5	.328	0.23	[-0.42;0.89]
Immature Reticulocytes [%]	7.15	4.58		6.78	2.36	174.5	.597	0.12	[-0.53;0.77]
Thrombocyte [10^9/L]	229.46	54.14		244.87	54.61	163.0	.405	-0.28	[-0.93;0.37]
**Inflammation/Immunology**									
SARS-CoV2 Spike Antibody [U/mL]	1546.23	2106.18		447.12	411.87	8.0	**.045**	0.65	[-0.26;1.55]
CH50 [2] [U/mL]	55.20	2.63		50.18	5.93	64.0	.068	0.90	[-0.22;1.99]
C-Reactive Protein [1] [mg/L]	0.80	0.75		0.54	.49	238.0	.167	0.45	[-0.21;1.10]
FT3 [pmol/L]	5.17	1.08		5.11	.60	223.5	.348	0.08	[-0.58;0.73]
FT4 [pmol/L]	16.22	3.24		15.78	2.59	191.5	.946	0.16	[-0.50;0.81]
Thyroid-stimulating hormone (TSH) [mlU/L]	1.59	0.68		1.78	.89	184.5	.791	-0.23	[-0.88;0.43]
IgA [g/L]	1.85	0.53		1.97	.82	181.0	.849	-0.16	[-0.81;0.50]
IgE [IU/mL]	141.07	260.09		193.13	444.55	218.0	.435	-0.13	[-0.78;0.52]
IgG [g/L]	11.95	2.79		10.52	1.55	254.0	.077	0.71	[0.04;1.38]
IgM [g/L]	1.04	0.46		0.96	.51	219.5	.406	0.16	[-0.50;0.81]
IL-1β [1] [pg/mL]	6.83	8.92		7.05	7.27	164.0	.419	-0.03	[-0.68;0.62]

IL-10 [1] [pg/mL]	2.92	5.28		2.54	3.87	163.0	.491	0.09	[-0.57;0.74]
IL- 6[1] [pg/mL]	1.45	0.85		1.03	.53	242.5	.083	0.66	[-0.02;1.32]
IL-8 [1] [ng/L]	3.65	1.56		3.58	1.99	216.0	.462	0.04	[-0.61;0.69]
Complement C3c [g/L]	1.08	0.12		1.06	.15	165.0	.621	0.15	[-0.53;0.83]
Complement C4 [g/L]	0.22	0.06		0.21	.06	172.5	.456	0.26	[-0.43;0.94]
TNF-α [pg/mL]	4.22	1.07		4.78	1.93	146.0	.253	-0.33	[-0.99;0.33]
LBP [µg/mL]	4.63	1.03		4.30	1.12	229.0	.276	0.30	[-0.36;0.95]
**Coagulation / Hormones**									
ALT [U/L]	25.77	7.87		31.40	14.97	168.5	.491	-0.42	[-1.08;0.24]
AST [U/L]	38.85	19.74		34.47	11.53	219.5	.525	0.30	[-0.35;0.96]
D-Dimers [1] [mg/IU]	0.17	0.17		0.14	.10	175.5	.778	0.23	[-0.45;0.90]
Fibrinogen [g/L]	2.56	0.29		2.47	.37	193.5	.460	0.24	[-0.44;0.92]
Folic acid [nmol/L]	18.76	7.40		17.71	8.23	169.5	.388	0.13	[-0.58;0.84]
Thrombin time [sec]	16.99	0.61		16.93	.75	179.0	.756	0.08	[-0.59;0.76]
PTT [sec]	29.06	1.79		30.56	3.23	124.0	.199	-0.52	[-1.20;0.17]

Quick [%] (Internal laboratory calculation – not externally comparable)	87.58	15.84		85.71	11.44	192.0	.487	0.15	[-0.53;0.82]
**Damage Markers**									
Calculated GFR CKD EPI [mL/min]	101.15	21.05		101.83	13.99	197.0	.968	-0.04	[-0.69;0.61]
CK [U/L]	605.92	974.41		228.13	153.49	224.5	.443	0.70	[0.02;1.36]
Urea [mmol/L]	5.53	1.83		5.46	1.50	191.5	.937	0.04	[-0.61;0.69]
**Uric Acid [µmol/L]**	279.54	57.84		333.23	63.67	99.0	**.012**	-0.87	[-1.54;-0.18]
Lactate Dehydrogenase (LDH) [U/L]	213.08	37.96		206.37	35.25	215.0	.606	0.19	[-0.47;0.84]
Myoglobin [µg/L]	116.15	262.57		37.85	19.84	193.0	.483	0.52	[-0.16;1.19]
NT pro BNP [pg/mL]	49.82	33.54		33.86	21.79	251.0	.144	0.62	[-0.05;1.28]
**Troponin-T[1] [ng/L]**	13.23	9.51		6.00	4.69	318.5	**.001**	1.12	[0.42;1.80]
**Electrolytes/Micronutrients**									
**Zinc [µmol/L]**	12.26	2.65		13.07	1.83	116.0	**.038**	-0.38	[-1.04;0.28]
Glucose [mg/dL	77.00	15.57		82.10	14.05	154.5	.290	-0.35	[-1.00;0.31]
**Potassium [mmol/L]**	4.03	0.39		4.37	.41	105.5	**.018**	-0.85	[-1.52;-0.17]

Natrium [mmol/L]	140.62	1.80		140.90	1.65	173.5	.572	-0.17	[-0.82;0.48]
Selen [µg/L]	80.68	13.54		80.29	12.83	19.0	.978	0.03	[-0.62;0.68]
**Metabolism/Vitamins**									
Vitamin B1 [nmol/L]	149.42	25.67		137.54	27.54	199.0	.368	0.44	[-0.25;1.12]
Vitamin B12 [pmol/L]	348.62	133.27		328.57	185.73	219.5	.300	0.12	[-0.54;0.77]
Vitamin B6 [nmol/L]	117.42	70.34		163.32	162.75	119.5	.157	-0.32	[-1.00;0.36]
**Vitamin D25OH [µg/L]**	34.69	11.15		26.48	12.86	27.5	**.026**	0.66	[-0.01;1.33]
Protein [g/L]	76.31	5.78		74.03	3.11	25.5	.092	0.55	[-0.11;1.22]
Creatinine [µmol/L]	84.77	18.89		84.73	1.43	182.5	.751	0.00	[-0.65;0.65]
Triglyceride [mmol/L]	1.07	0.75		1.29	.72	131.5	.160	-0.30	[-0.96;0.37]
Cholesterol [mmol/L]	4.55	1.32		4.44	.88	172.0	.790	0.10	[-0.56;0.76]

[1] Concentrations lower limit of detection were replaced by half of the detection limit (LOD)

[2] 12 individuals with CH50 concentrations > 60 were excluded from the analysis of CH50. 4 individuals with folic acid concentrations > 45.4 were excluded from the analysis of folic acid.

Two-tailed Man-Whitney U- Test. Cohen's *d* is reported as effect size measure [95%-CI of *d*]
