# Supplementary material for: Blood Profiling of Athletes after COVID-19: Differences in Blood Profiles of Post-COVID-19 Athletes Compared to Uninfected Athletic Individuals—An Exploratory Analysis
Source: Biomedicines. 2023 Jul 6;11(7):1911. doi: 10.3390/biomedicines11071911 (PMC10377547; doi:10.3390/biomedicines11071911)
Supplement: Supplementary file 1 [file biomedicines-11-01911-s001.zip › Table S3.pdf]

Table S3

Differences between ATH and HC controlled for age

		b	SE	95%-CI[1]	*t*	*df*	*p*
**Blood cell (count)**							
Basophile absolute [10^9/L]	(Intercept)	0.03	0.01	[0.02; 0.05]	3.91	87	0.00
	Age	0.00	0.00	[0.00; 0.00]	0.79	87	0.43
	group	-0.01	0.01	[-0.02; 0.01]	-1.04	87	0.30
Basophile relative [%]	(Intercept)	0.63	0.11	[0.42; 0.84]	5.95	87	0.00
	Age	0.00	0.00	[0.00; 0.01]	0.34	87	0.73
	group	-0.07	0.07	[-0.20; 0.07]	-0.95	87	0.35
**Eosinophile absolute [10^9/L]**	(Intercept)	0.07	0.02	[0.04; 0.10]	4.59	87	0.00
	Age	0.00	0.00	[0.00; 0.00]	2.00	87	0.05
	group	-0.02	0.01	[-0.04; 0.00]	-1.62	87	0.11
Eosinophile relative [%]	(Intercept)	1.32	0.33	[0.67; 1.98]	4.01	87	0.00

	Age	0.01	0.01	[-0.01; 0.03]	1.01	87	0.32
	group	-0.03	0.22	[-0.46; 0.40]	-0.15	87	0.88
Erythropoietin [mIU/ml]	(Intercept)	10.17	1.30	[7.58; 12.76]	7.81	85	0.00
	Age	0.02	0.03	[-0.05; 0.09]	0.48	85	0.63
	group	-0.69	0.85	[-2.37; 0.99]	-0.82	85	0.42
**Red blood cell distribution width (RDW) [%]**	(Intercept)	11.90	0.21	[11.49; 12.31]	57.47	87	0.00
	Age	0.02	0.01	[0.00; 0.03]	2.82	87	0.01
	group	0.17	0.14	[-0.10; 0.44]	1.27	87	0.21
Erythrocyte [10^{12}/L]	(Intercept)	4.57	0.19	[4.19; 4.94]	23.98	87	0.00
	Age	0.00	0.01	[-0.01; 0.01]	0.24	87	0.81
	group	0.12	0.12	[-0.12; 0.36]	0.98	87	0.33
Ferritin [µg/L]	(Intercept)	39.14	25.34	[-11.23; 89.51]	1.54	87	0.13
	Age	1.34	0.71	[-0.08; 2.75]	1.88	87	0.06
	group	5.28	16.84	[-28.18; 38.75]	0.31	87	0.75
**Hb content reticulocytes [pg]**	(Intercept)	33.55	0.49	[32.57; 34.52]	68.68	87	0.00

	Age	0.01	0.01	[-0.01; 0.04]	1.10	87	0.28
	group	-0.62	0.32	[-1.26; 0.01]	-1.95	87	**0.05**
Hematocrit [%]	(Intercept)	0.40	0.01	[0.37; 0.43]	29.85	87	0.00
	Age	0.00	0.00	[0.00; 0.00]	1.00	87	0.32
	group	0.00	0.01	[-0.01; 0.02]	0.44	87	0.66
Hemoglobin [g/dL]	(Intercept)	13.75	0.47	[12.82; 14.68]	29.34	87	0.00
	Age	0.01	0.01	[-0.02; 0.03]	0.53	87	0.60
	group	0.03	0.31	[-0.58; 0.65]	0.11	87	0.92
**Leukocyte [10^9/L]**	(Intercept)	5.85	0.45	[4.95; 6.75]	12.94	87	0.00
	Age	0.02	0.01	[-0.01; 0.04]	1.31	87	0.20
	group	-0.71	0.30	[-1.30; -0.13]	-2.42	87	**0.02**
**Soluble Transferrin receptor [mg/L]**	(Intercept)	3.41	0.29	[2.82, 3.99]	11.56	87	0.00
	Age	0.00	0.00	[-0.01, 0.02]	0.53	87	0.60
	group	-0.79	0.19	[-1.17, -0.40]	-4.05	87	**0.00**
**Lymphocyte absolute [10^9/L]**	(Intercept)	2.14	0.16	[1.83; 2.45]	13.72	87	**0.00**

	Age	0.00	0.00	[-0.01; 0.00]	-0.91	87	0.366
	group	-0.19	0.10	[-0.39; 0.02]	-1.83	87	0.070
**Lymphocyte relative [%]**	(Intercept)	36.62	2.52	[31.61; 41.63]	14.52	87	0.00
	Age	-0.16	0.07	[-0.30; -0.03]	-2.35	87	0.02
	group	0.95	1.65	[-2.33; 4.23]	0.57	87	0.57
**MCH [pg]**	(Intercept)	30.10	0.36	[29.38; 30.82]	83.10	87	0.00
	Age	0.01	0.01	[-0.01; 0.03]	0.66	87	0.51
	group	-0.56	0.24	[-1.04; -0.08]	-2.33	87	**0.02**
**MCHC [g/dL]**	(Intercept)	34.49	0.34	[33.81; 35.17]	100.72	87	**0.00**
	Age	-0.02	0.00	[-0.04; 0.00]	-2.24	87	**0.027**
	group	-0.36	0.22	[-0.79; 0.08]	-1.63	87	0.106
**MCV [fl]**	(Intercept)	86.08	1.42	[83.26; 88.90]	60.70	87	0.00
	Age	0.09	0.04	[0.01; 0.16]	2.33	87	0.02
	group	-0.72	0.94	[-2.60; 1.15]	-0.77	87	0.45
Monocyte absolute [10^9/L]	(Intercept)	0.39	0.06	[0.28; 0.50]	6.83	87	0.00

	Age	0.00	0.00	[0.00; 0.01]	1.67	87	0.10
	group	-0.01	0.04	[-0.08; 0.06]	-0.26	87	0.80
Monocyte relative [%]	(Intercept)	6.98	0.71	[5.57; 8.39]	9.84	87	0.00
	Age	0.02	0.02	[-0.02; 0.05]	0.89	87	0.37
	group	0.56	0.46	[-0.36; 1.49]	1.21	87	0.23
MTV [fl]	(Intercept)	10.44	0.57	[9.31; 11.58]	18.35	87	0.00
	Age	0.00	0.02	[-0.03; 0.03]	-0.02	87	0.99
	group	0.49	0.37	[-0.24; 1.22]	1.33	87	0.19
**Neutrophile absolute [10^9/L]**	(Intercept)	3.16	0.32	[2.53; 3.79]	9.98	87	0.00
	Age	0.02	0.01	[0.00; 0.03]	1.87	87	0.06
	group	-0.50	0.21	[-0.91; -0.09]	-2.41	87	**0.02**
Neutrophile relative [%]	(Intercept)	52.72	2.98	[46.80; 58.64]	17.71	87	0.00
	Age	0.15	0.08	[-0.01; 0.31]	1.88	87	0.06
	group	-0.67	1.95	[-4.54; 3.20]	-0.34	87	0.73
Reticulocyte absolute [10^9/L]	(Intercept)	58.27	6.24	[45.87, 70.66]	9.34	87	0.00

	Age	0.13	0.17	[-0.20, 0.47]	0.81	87	0.420
	group	-5.81	4.09	[-13.94, 2.31]	-1.42	87	0.158
Reticulocyte relative [%]	(Intercept)	1.31	0.13	[1.05; 1.58]	9.98	87	0.00
	Age	0.00	0.00	[-0.01; 0.01]	0.46	87	0.64
	group	-0.15	0.09	[-0.32; 0.03]	-1.68	87	0.10
Immature Reticulocytes [%]	(Intercept)	5.51	0.93	[3.66; 7.36]	5.91	87	0.00
	Age	0.03	0.02	[-0.02; 0.08]	1.33	87	0.19
	group	0.17	0.61	[-1.05; 1.38]	0.27	87	0.79
Thrombocyte [10^9/L]	(Intercept)	251.31	19.47	[212.62; 290.00]	12.91	87	0.00
	Age	0.17	0.52	[-0.87; 1.21]	0.33	87	0.74
	group	-11.10	12.52	[-35.98; 13.78]	-0.89	87	0.38
**Inflammation/Immunology**							
**SARS-CoV2 Spike Antibody [U/mL]**	(Intercept)	1945.33	478.02	[981.94; 2908.72]	4.07	44	0.00
	Age	-13.15	13.11	[-39.58; 13.27]	-1.00	44	0.32
	group	-898.88	337.97	[-1580.01; -217.76]	-2.66	44	**0.01**

CH50 [2] [U/mL]	(Intercept)	51.06	3.35	[44.30; 57.83]	15.23	42	0.00
	Age	0.11	0.09	[-0.06; 0.28]	1.27	42	0.21
	group	-4.03	2.15	[-8.38; 0.32]	-1.87	42	0.07
FT3 [pmol/L]	(Intercept)	5.45	0.24	[4.98; 5.92]	22.84	86	0.00
	Age	-0.01	0.01	[-0.02; 0.00]	-1.47	86	0.14
	group	-0.25	0.16	[-0.55; 0.06]	-1.58	86	0.12
FT4 [pmol/L]	(Intercept)	16.32	0.91	[14.50; 18.13]	17.90	86	0.00
	Age	-0.02	0.02	[-0.07; 0.03]	-0.84	86	0.40
	group	-0.49	0.59	[-1.67; 0.69]	-0.83	86	0.41
**IgA [g/L]**	(Intercept)	1.12	0.25	[0.63; 1.61]	4.52	84	0.00
	Age	0.02	0.01	[0.00; 0.03]	2.57	84	0.01
	group	0.04	0.16	[-0.27; 0.35]	0.27	84	0.79
IgE [IU/mL]	(Intercept)	45.21	14.14	[17.10; 73.31]	3.20	86	0.00
	Age	-0.12	0.36	[-0.83; 0.59]	-0.34	86	0.74
	group	-13.15	8.92	[-30.87; 4.58]	-1.47	86	0.14

IgG [g/L]	(Intercept)	10.68	0.79	[9.11; 12.25]	13.49	85	0.00
	Age	0.02	0.02	[-0.02; 0.06]	0.92	85	0.36
	group	-0.80	0.52	[-1.83; 0.23]	-1.55	85	0.13
IgM [g/L]	(Intercept)	1.27	0.22	[0.83; 1.70]	5.76	85	0.00
	Age	0.00	0.01	[-0.01; 0.01]	-0.39	85	0.70
	group	-0.18	0.14	[-0.46; 0.10]	-1.27	85	0.21
**Complement C3c [g/L]**	(Intercept)	1.10	0.06	[0.98; 1.22]	18.63	73	0.00
	Age	0.00	0.00	[0.00; 0.00]	0.55	73	0.59
	group	-0.09	0.04	[-0.16; -0.01]	-2.34	73	**0.02**
**Complement C4 [g/L]**	(Intercept)	0.16	0.02	[0.12; 0.20]	8.11	73	0.00
	Age	0.00	0.00	[0.00; 0.00]	3.02	73	0.00
	group	-0.02	0.01	[-0.04; 0.01]	-1.45	73	0.15
TNF-α [pg/mL]	(Intercept)	4.20	0.49	[3.22; 5.17]	8.56	84	0.00
	Age	0.00	0.01	[-0.02; 0.03]	0.10	84	0.92
	group	-0.22	0.33	[-0.88; 0.43]	-0.69	84	0.49

LBP [µg/mL]	(Intercept)	4.23	0.49	[3.25; 5.21]	8.56	85	0.00
	Age	0.01	0.01	[-0.01; 0.04]	1.00	85	0.32
	group	-0.54	0.31	[-1.16; 0.07]	-1.75	85	0.08
**Coagulation**							
**Fibrinogen [g/L]**	(Intercept)	2.20	0.13	[1.93; 2.46]	16.52	82	0.00
	Age	0.01	0.00	[0.01; 0.02]	4.03	82	0.00
	group	-0.11	0.08	[-0.28; 0.06]	-1.33	82	0.19
**Folic Acid [nmol/L]**	(Intercept)	10.55	2.81	[4.94; 16.15]	3.75	75	0.00
	Age	0.28	0.08	[0.12; 0.43]	3.60	75	0.00
	group	-1.55	1.83	[-5.20; 2.11]	-0.84	75	0.40
**Thrombin time [sec]**	(Intercept)	17.96	0.29	[17.39; 18.53]	62.63	82	0.00
	Age	-0.03	0.01	[-0.04; -0.01]	-3.46	82	0.00
	group	-0.04	0.18	[-0.41; 0.32]	-0.23	82	0.82
PTT [sec]	(Intercept)	27.87	0.99	[25.89; 29.85]	28.02	82	0.00
	Age	0.01	0.03	[-0.04; 0.07]	0.48	82	0.63

	group	1.20	0.63	[-0.04; 2.44]	1.92	82	0.06
**Quick [%] (Internal laboratory calculation – not externally comparable)**	(Intercept)	90.75	5.55	[79.70; 101.81]	16.34	81	0.00
	Age	0.23	0.15	[-0.08; 0.53]	1.49	81	0.14
	group	-8.76	3.50	[-15.73; -1.79]	-2.50	81	**0.01**
**Damage Markers**							
**Calculated GFR CKD EPI [mL/min]**	(Intercept)	133.27	5.09	[123.16; 143.38]	26.20	87	0.00
	Age	-0.79	0.13	[-1.06; -0.52]	-5.89	87	0.00
	group	-5.97	3.31	[-12.55; 0.61]	-1.80	87	0.08
**CK [U/L]**	(Intercept)	92.07	22.26	[47.83; 136.31]	4.14	87	0.00
	Age	-0.05	0.59	[-1.22; 1.13]	-0.08	87	0.93
	group	32.50	15.20	[2.28; 62.72]	2.14	87	**0.04**
**Urea [mmol/L]**	(Intercept)	3.15	0.44	[2.28; 4.03]	7.14	87	0.00
	Age	0.04	0.01	[0.01; 0.06]	2.94	87	0.00
	group	0.14	0.28	[-0.42; 0.70]	0.50	87	0.62
Uric Acid [µmol/L]	(Intercept)	227.86	25.87	[176.44; 279.29]	8.81	87	0.00

	Age	1.01	0.69	[-0.37; 2.38]	1.46	87	0.15
	group	19.81	16.67	[-13.32; 52.95]	1.19	87	0.24
Lactate Dehydrogenase (LDH) [U/L]	(Intercept)	179.63	13.26	[153.27; 205.99]	13.55	87	0.00
	Age	0.48	0.35	[-0.22; 1.18]	1.35	87	0.18
	group	-3.28	8.69	[-20.54; 13.99]	-0.38	87	0.71
Myoglobin [µg/L]	(Intercept)	25.60	5.09	[15.41; 35.78]	5.03	61	0.00
	Age	0.09	0.14	[-0.18; 0.36]	0.67	61	0.50
	group	2.88	3.54	[-4.20; 9.96]	0.81	61	0.42
NT pro BNP [pg/mL]	(Intercept)	41.09	9.98	[21.24; 60.93]	4.12	86	0.00
	Age	0.32	0.27	[-0.21; 0.85]	1.20	86	0.23
	group	-9.20	6.62	[-22.37; 3.96]	-1.39	86	0.17
**Electrolytes/Micronutrients**							
**Zinc [µmol/L]**	(Intercept)	11.23	0.64	[9.95; 12.50]	17.46	86	0.00
	Age	0.01	0.02	[-0.03; 0.04]	0.31	86	0.76
	group	0.95	0.42	[0.11; 1.79]	2.24	86	**0.03**

**Glucose [mg/dL]**	(Intercept)	70.11	3.86	[62.44; 77.78]	18.17	87	0.00
	Age	0.24	0.10	[0.05; 0.44]	2.45	87	0.02
	group	4.02	2.50	[-0.96; 8.99]	1.60	87	0.11
**Potassium [mmol/L]**	(Intercept)	3.57	0.13	[3.32; 3.82]	27.97	87	0.00
	Age	0.01	0.00	[0.00; 0.02]	3.02	87	0.00
	group	0.39	0.08	[0.23; 0.55]	4.81	87	**0.00**
**Sodium [mmol/L]**	(Intercept)	139.86	0.67	[138.52; 141.19]	208.11	87	0.00
	Age	-0.01	0.02	[-0.05; 0.03]	-0.52	87	0.60
	group	0.91	0.44	[0.04; 1.79]	2.08	87	**0.04**
Selen [µg/L]	(Intercept)	71.05	5.06	[60.99; 81.11]	14.05	84	0.00
	Age	0.20	0.14	[-0.07; 0.47]	1.50	84	0.14
	group	-2.32	3.29	[-8.86; 4.22]	-0.70	84	0.48
**Vitamins/Metabolism**							
Vitamin B1 [nmol/L]	(Intercept)	137.24	11.40	[114.56; 159.91]	12.04	84	0.00
	Age	0.16	0.31	[-0.46; 0.77]	0.51	84	0.61

	group	-2.22	7.32	[-16.78; 12.34]	-0.30	84	0.76
Vitamin B12 [pmol/L]	(Intercept)	247.82	37.63	[172.99,322.66]	6.59	83	0.00
	Age	1.03	1.01	[-0.97; 3.04]	1.02	83	0.31
	group	6.30	24.21	[-41.86; 54.46]	0.26	83	0.80
**Vitamin B6 [nmol/L]**	(Intercept)	101.28	12.67	[76.08; 126.49]	7.99	84	0.00
	Age	-0.13	0.35	[-0.82; 0.56]	-0.37	84	0.71
	group	19.55	8.23	[3.19; 35.91]	2.38	84	**0.02**
**Vitamin D25OH**	(Intercept)	36.03	3.69	[28.70; 43.36]	9.77	86	0.00
	Age	-0.07	0.10	[-0.27; 0.13]	-0.70	86	0.48
	group	-8.81	2.34	[-13.46; -4.15]	-3.76	86	**0.00**
ALT [U/L]	(Intercept)	17.74	3.63	[0.53; 24.95]	4.89	86	0.00
	Age	0.06	0.10	[-0.13; 0.26]	0.63	86	0.53
	group	1.97	2.39	[-2.78; 6.72]	0.82	86	0.41
AST [U/L]	(Intercept)	27.14	3.44	[20.29; 33.98]	7.88	87	0.00
	Age	-0.06	0.09	[-0.24; 0.13]	-0.60	87	0.55

	group	2.72	2.29	[-1.83; 7.27]	1.19	87	0.24
**Protein [g/L]**	(Intercept)	77.41	1.54	[74.34; 80.47]	50.17	86	0.00
	Age	-0.03	0.04	[-0.12; 0.05]	-0.79	86	0.43
	group	-2.80	0.99	[-4.77; -0.84]	-2.84	86	**0.01**
Thyroid-stimulating hormone (TSH) [mlU/L]	(Intercept)	1.43	0.27	[0.89; 1.96]	5.34	87	0.00
	Age	0.00	0.01	[-0.01; 0.02]	0.13	87	0.90
	group	0.24	0.17	[-0.11; 0.59]	1.37	87	0.17
Creatinine [µmol/L]	(Intercept)	65.63	5.33	[55.02; 76.23]	12.30	87	0.00
	Age	0.14	0.14	[-0.14; 0.42]	1.00	87	0.32
	group	6.68	3.46	[-0.20; 13.55]	1.93	87	0.06
**Triglyceride [mmol/L]**	(Intercept)	0.43	0.14	[0.15; 0.72]	3.01	84	0.00
	Age	0.01	0.00	[0.00; 0.02]	2.77	84	0.01
	group	0.18	0.10	[-0.01; 0.37]	1.89	84	0.06
**Cholesterol [mmol/L]**	(Intercept)	3.36	0.29	[2.79; 3.94]	11.72	84	0.00
	Age	0.04	0.01	[0.02; 0.05]	4.78	84	**0.00**

	group	-0.07	0.18	[-0.43; 0.30]	-0.37	84	0.71

Note. Results of robust linear regression models (R package: "Robust"). *p* - values < .05 were considered as significant. Significant values for group are given in bold. No significant age difference between ATH and HC (**ATH:** $M = 34.6$, SD = 12.24; **HC:** $M = 31.9$, SD = 10.59; $W = 820.5$, $p = .477$)

[1] 95% CI of regression parameter *b*
